# Determination of sample size for a multi-class classifier based on single-nucleotide polymorphisms: a volume under the surface approach

**DOI:** 10.1186/1471-2105-15-190

**Published:** 2014-06-14

**Authors:** Xinyu Liu, Yupeng Wang, TN Sriram

**Affiliations:** 1 Department of Statistics, University of Georgia, Athens, GA 30602, USA; 2 Computational Biology Service Unit, Cornell University, Ithaca, NY 14853, USA

**Keywords:** Area under the receiver operating characteristic curve, Classification, HapMap data, Heterogeneous stock mice data, Probability of correct classification, Receiver operating characteristic, Sample size determination

## Abstract

**Background:**

Data on single-nucleotide polymorphisms (SNPs) have been found to be useful in predicting phenotypes ranging from an individual’s class membership to his/her risk of developing a disease. In multi-class classification scenarios, clinical samples are often limited due to cost constraints, making it necessary to determine the sample size needed to build an accurate classifier based on SNPs. The performance of such classifiers can be assessed using the Area Under the Receiver Operating Characteristic (*ROC*) Curve (*AUC*) for two classes and the Volume Under the *ROC* hyper-Surface (*VUS*) for three or more classes. Sample size determination based on *AUC* or *VUS* would not only guarantee an overall correct classification rate, but also make studies more cost-effective.

**Results:**

For coded SNP data from *D*(≥2) classes, we derive an optimal Bayes classifier and a linear classifier, and obtain a normal approximation to the probability of correct classification for each classifier. These approximations are then used to evaluate the associated *AUCs* or *VUSs*, whose accuracies are validated using Monte Carlo simulations. We give a sample size determination method, which ensures that the difference between the two approximate *AUCs* (or *VUSs*) is below a pre-specified threshold. The performance of our sample size determination method is then illustrated via simulations. For the *HapMap* data with three and four populations, a linear classifier is built using 92 independent SNPs and the required total sample sizes are determined for a continuum of threshold values. In all, four different sample size determination studies are conducted with the *HapMap* data, covering cases involving *well-separated* populations to *poorly-separated* ones.

**Conclusion:**

For multi-classes, we have developed a sample size determination methodology and illustrated its usefulness in obtaining a required sample size from the estimated learning curve. For classification scenarios, this methodology will help scientists determine whether a sample at hand is adequate or more samples are required to achieve a pre-specified accuracy. A PDF manual for R package “SampleSizeSNP” is given in Additional file 1, and a ZIP file of the R package “SampleSizeSNP” is given in Additional file 2.

## Background

Data on single-nucleotide polymorphisms (SNPs) have been found to be useful in predicting an individual’s class membership or his/her response to a drug, susceptibility to environmental factors such as toxins, and the risk of developing a particular disease, among others [1-5]. The classification literature provides a variety of classifiers (e.g., Support Vector Machine, genetic programming, Neural Networks and Logistic Regression) and sample size determination methods [6-10], but most of these are only applicable to continuous data.

Recently Liu *et al*. [11] developed an optimal Bayes classifier and a linear classifier for coded SNP data from two classes, and obtained a normal approximation to the probability of correct classification (*PCC*) for each classifier. They also proposed a sample size determination methodology to determine an adequate sample size, which ensures that the difference between the two approximate *PCCs* is below a pre-specified threshold value. Using Monte Carlo simulations, Liu *et al*. [11] assessed the validity of their approximations. Furthermore, they illustrated the performance of their sample size determination method via simulations and a real data analysis using the *HapMap* data on two populations—Chinese and Japanese.

While Liu *et al*. [11] showed that their sample size determination method is competitive, they also pointed out that an additional maximization step is required in order to determine the discrimination values for each of their classifiers; see their REMARK1 in their article for more details. When there are three or more classes, however, determination of such discrimination values is not only more difficult, but also increases the overall computational burden. In a two-class scenario, a well known way to overcome this difficulty is to consider the Receiver Operating Characteristic (*ROC*) curve, which plots the True Positive Rates vs. False Positives Rates, at various discrimination values [12,13]. Note that the *ROC* allows the discrimination value to be varied and it simultaneously explores all possible combinations of the correct classification rates [14]. The Area Under the *ROC* curve (*AUC*) is commonly used as a scalar performance measure, which allows classifiers to be compared independent of the discrimination values. Unfortunately, the *AUC* measure is only applicable to a two-class scenario. A popular extension of the *AUC* measure, known as the Volume Under the *ROC* hyper-Surface (*VUS*) measure, is often used in a multi-class scenario (see e.g., Landgrebe and Duin [14] and Landgrebe and Paclik 2010 [15]).

This article revisits the problem of sample size determination in classification scenarios involving coded SNP data, but uses the *AUC* and the *VUS* as performance measures for two-class and multi-class scenarios, respectively. More specifically, for coded SNP data from *D*(≥2) classes, we derive an optimal Bayes classifier and obtain a normal approximation to its probability of correct classification, which is denoted by *PCC*(*∞*). We also derive a linear classifier and obtain a normal approximation to its probability of correct classification, which is denoted by $\text{PCC}(\vec{n})$. For an overall assessment of each of the classifiers, we define the scalar measures *AUC* (for two-class) and *VUS* (for multi-class), and correspondingly define the quantities $\text{AUC}(\infty ),\text{AUC}(\vec{n}),\text{VUS}(\infty )\;\text{and}\;\text{VUS}(\vec{n})$ for each classification scenario. For the two-class scenario, we propose to determine the sample size *n* for which $\text{AUC}(\infty )-\text{AUC}(\vec{n})<\gamma$, where *γ*∈(0,1) is a pre-specified threshold value. Whereas, for the multi-class scenario, we propose to determine the sample size *n* for which $\text{VUS}(\infty )-\text{VUS}(\vec{n})<\gamma$. A computational method to determine the total sample size for various values of *γ* is described. Monte Carlo simulations are carried out to corroborate our theoretical approximations, and the performance of our sample size determination method is assessed via simulations and analysis of the *HapMap* data consisting of 3 and 4 populations, respectively. In all, four different sample size determination studies are conducted with the *HapMap* data, covering cases involving *well-separated* populations to *poorly-separated* ones. Details are given in the data analysis section.

R software was used to carry out all the computations. A PDF manual for R package “SampleSizeSNP” is given in Additional file 1, and a ZIP file of the R package “SampleSizeSNP” is given in Additional file 2.

## Methods

### Assumptions

Suppose there are *D*(≥2) distinct classes denoted by *C*_1_,…,*C*_*D*_, consisting of *n*_1_,…,*n*_*D*_ subjects, respectively. For each subject, we observe a *p*-dimensional SNP vector, $\vec{x}={\left(x_{1},x_{2},\mathrm{..},x_{p}\right)}^{\prime }$, where typically *p* is much larger (>>) than $\overset{D}{\underset{i=1}{\sum }}n_{i}$, and the *j*th SNP is coded in such a way *x*_*j*_=0,1,2, which denotes the number of minor alleles in the genotype “aa”, “Aa” and “AA”, respectively. It is possible that some of the SNPs are highly correlated, leading us to choose one SNP to represent a set of highly correlated ones. For classification and sample size determination, we make the following assumptions: 

1. For an *m* such that $\overset{D}{\underset{i=1}{\sum }}n_{i}<<m<p$, the data vector $\vec{x}={\left(x_{1},\ldots ,x_{m}\right)}^{\prime }$ consists only of *m* SNPs, which are statistically independent. That is, the rest of the (*p*−*m*) correlated SNPs are not used for classification.

2. For each *k*=1,…,*D* and *j*=1,…,*m*, we postulate Hardy-Weinberg equilibrium, according to which the probability mass function of the coded SNP (*X*_*j*_) belonging to class *k* is given by

Pk(Xj=xj|θk,j)=2xjθk,jxj(1−θk,j)2−xj,xj=0,1,2,

where *θ*_*k*,*j*_ is the minor allele frequency at locus *j* in class *k*, and by definition *θ*_*k*,*j*_∈(0.01,0.5). Here, *θ*_*k*,*j*_<0.5 because it is the minor allele frequency, and *θ*_*k*,*j*_>0.01 ensures that the polymorphism is not a *mutation*. For each *k*=1,…,*D*, let ${\vec{\theta }}_{k}={\left(\theta_{k,1},\ldots ,\theta_{k,m}\right)}^{\prime }$ denote the parameter vector corresponding to the class *C*_*k*_.

3. There is a percentage *ρ* of the *m* SNPs with marginal effect on any two classes, and let *l*=⌊*ρm*⌋ be the number of SNPs with marginal effects.

### The optimal classifier and its ***PCC***

By the assumptions above, the conditional mass function of $\vec{X}={\left(X_{1},\ldots ,X_{l}\right)}^{\prime }$ given the class *C*_*k*_, *k*=1,…,*D*, is

fk(X→=x→|θ→k)=∏j=1l2xjθk,jxj(1−θk,j)2−xj.

Suppose $\pi_{k}=P(\vec{x}\in C_{k})$ and we denote the marginal mass function $f(\vec{x})=\overset{D}{\underset{k=1}{\sum }}\pi_{k}f_{k}(\vec{x}|{\vec{\theta }}_{k})$, then for each 1≤*k*≤*D*, the posterior mass function of the class *C*_*k*_ given $\vec{x}$ is

$$\begin{array}{l} \tau_{k}({\vec{\theta }}_{k}|\vec{x})=\frac{\pi_{k}f_{k}(\vec{x}|{\vec{\theta }}_{k})}{f(\vec{x})}. \end{array}$$

For any fixed *k*=1,…,*D*, the Bayes classification rule then classifies $\vec{x}$ to the class *C*_*k*_ if

(1) $$\begin{array}{l} \frac{\tau_{k}({\vec{\theta }}_{k}|\vec{x})}{\tau_{k^{\prime }}({\vec{\theta }}_{k^{\prime }}|\vec{x})}>1 \end{array}$$

for all *k*^′^≠*k*. This leads to the *optimal* Bayes classifier, which classifies $\vec{x}$ to *C*_*k*_ if

(2) $$\begin{array}{l} \overset{l}{\underset{j=1}{\sum }}b_{k,k^{\prime }}^{j}x_{j}>K_{k,k^{\prime }} \end{array}$$

for all *k*^′^≠*k*, where

(3) $$\begin{array}{lll} b_{k,k^{\prime }}^{j} & =\log\left(\frac{\theta_{k,j}(1-\theta_{k^{\prime },j})}{\theta_{k^{\prime },j}(1-\theta_{k,j})}\right)\;\;\text{and}\;\;K_{k,k^{\prime }}\; & \;\\ & =\log\left(\frac{\pi_{k^{\prime }}}{\pi_{k}}\right)+2\log\left(\frac{1-\theta_{k^{\prime },j}}{1-\theta_{k,j}}\right).\; \end{array}$$

Then, the *PCC* of the optimal Bayes classifier is defined as

$$\begin{array}{l} \begin{array}{c} \text{PCC}(\infty )=\overset{D}{\underset{k=1}{\sum }}\pi_{k}P\left(\underset{k^{\prime }\neq k}{⋂}\left\{\overset{l}{\underset{j=1}{\sum }}b_{k,k^{\prime }}^{j}x_{j}>K_{k,k^{\prime }}\right\}|\vec{X}\in C_{k}\right). \end{array} \end{array}$$

In Additional file 3: Appendix 1, we derive a normal approximation for *PCC*(*∞*), as *l*→*∞*. That is, for large *l*, we show that

(4) $$\begin{array}{l} \text{PCC}(\infty )\approx \overset{D}{\underset{k=1}{\sum }}\pi_{k}\overset{\infty }{\underset{{\vec{K}}_{k}}{\int }}\phi \left(\vec{x};{\vec{\mu }}_{l,k},\Sigma_{l,k}\right)d\vec{x}, \end{array}$$

where ***ϕ*** is the (*D*−1)-dimensional multivariate normal density, $\overset{\infty }{\underset{{\vec{K}}_{k}}{\int }}$ is a multiple integral, ${\vec{K}}_{k}$ and ${\vec{\mu }}_{l,k}$ are (*D*−1)×1 vectors, and ***Σ***_*l*,*k*_ is a (*D*−1)×(*D*−1) matrix. All these quantities are defined in Additional file 3: Appendix 1.

In Additional file 3: Appendix 4, we give an expression for (4) for the case *D*=3.

### A linear classifier and its PCC

Motivated by the form of the optimal Bayes classifier in (2), we consider the following linear classifier that classifies $\vec{x}$ to the class *C*_*k*_ if

(5) $$\begin{array}{l} \overset{m}{\underset{j=1}{\sum }}{\hat{b}}_{k,k^{\prime }}^{j}w_{j,n}(k,k^{\prime })x_{j}>{\tilde{K}}_{k,k^{\prime }} \end{array}$$

for all *k*^′^≠*k*, where ${\hat{b}}_{k,k^{\prime }}^{j}=\log(\frac{{\hat{\theta }}_{k,j}(1-{\hat{\theta }}_{k^{\prime },j})}{{\hat{\theta }}_{k^{\prime },j}(1-{\hat{\theta }}_{k,j})})$, ${\hat{\theta }}_{k,j}\;\text{and}\;{\hat{\theta }}_{k^{\prime },j}$ are the maximum likelihood estimators of $\theta_{k,j}\;\text{and}\;\theta_{k^{\prime },j}$, respectively. Also, the values of the weights *w*_*j*,*n*_(*k*,*k*^′^) in (5) are determined in the following way: For each *j*=1,…,*m* and *k*^′^≠*k*, suppose we test the hypothesis $H_{0,j}^{k,k^{\prime }}:\theta_{k,j}=\theta_{k^{\prime },j}$ versus $H_{1,j}^{k,k^{\prime }}:\theta_{k,j}\neq \theta_{k^{\prime },j}$. Then *w*_*j*,*n*_(*k*,*k*^′^)=1 if $H_{0,j}^{k,k^{\prime }}$ is rejected; else *w*_*j*,*n*_(*k*,*k*^′^)=0. In Additional file 3: Appendix 2, we use the large sample theory to derive a Wald test of level *α* to test $H_{0,j}^{k,k^{\prime }}$ versus $H_{1,j}^{k,k^{\prime }}$, and an expression for the power, $1-\beta_{j}^{k,k^{\prime }}(n_{k},n_{k^{\prime }},h_{j})$, of this test, when $\theta_{k,j}-\theta_{k^{\prime },j}=h_{j}$.

In Additional file 3: Appendix 3, we derive a normal approximation for the *PCC* of the linear classifier, denoted by $\text{PCC}(\vec{n})$. That is, for large *l*, we show that

(6) $$\begin{array}{l} \text{PCC}(\vec{n})\approx \overset{D}{\underset{k=1}{\sum }}\pi_{k}\overset{\infty }{\underset{{\tilde{\vec{K}}}_{k}}{\int }}\phi \left(\vec{x};{\tilde{\vec{\mu }}}_{l,k},{\tilde{\Sigma }}_{l,k}\right)d\vec{x} \end{array}$$

Note that $\text{PCC}(\vec{n})$ depends on $\vec{n}={\left(n_{1},\ldots ,n_{D}\right)}^{\prime }$ through $\left({\tilde{\vec{\mu }}}_{m,k},{\tilde{\Sigma }}_{m,k}\right)$; see Additional file 3: Appendix 3 for details. In Additional file 3: Appendix 4, we give an expression for (6) for the case *D*=3.

### ***AUC*** and ***VUS*** for the optimal and linear classifiers

For any (*k*,*k*^′^), define

$$\begin{array}{l} \xi_{k,k^{\prime }}=P\left(\text{Classify}\;\vec{X}\;\text{to}\;C_{k^{\prime }}|\vec{X}\in C_{k}\right). \end{array}$$

Then, for the optimal Bayes classifier in (2) we have from (4) that

(7) $$\begin{array}{l} \xi_{k,k}\approx \overset{\infty }{\underset{{\vec{K}}_{k}}{\int }}\phi \left(\vec{x};{\vec{\mu }}_{l,k},\Sigma_{l,k}\right)d\vec{x} \end{array}$$

and similarly, for the linear classifier in (5), we have from (6) that

(8) $$\begin{array}{l} {\tilde{\xi }}_{k,k}\approx \overset{\infty }{\underset{{\tilde{\vec{K}}}_{k}}{\int }}\phi \left(\vec{x};{\tilde{\vec{\mu }}}_{l,k},{\tilde{\Sigma }}_{l,k}\right)d\vec{x}, \end{array}$$

for *k*=1,…,*D*. When *D*=2, for the optimal Bayes classifier, the *ROC*(*∞*) for two classes is the curve *ξ*_2,2_ vs. (1−*ξ*_1,1_). Then, the *AUC*(*∞*) is

$$\begin{array}{lll} \text{AUC}(\infty ) & =\int \xi_{2,2}d\xi_{1,1}.\; & \; \end{array}$$

However, when the number of classes *D*≥3, we need to consider the volume under the *ROC* hypersurface. Following the work of Landgrebe and Duin [14], the *VUS* is defined as

(9) $$\begin{array}{l} \begin{array}{c} \text{VUS}(\infty )=\int \ldots \int \xi_{D,D}d\xi_{1,1}\xi_{2,2}\ldots \xi_{\left(D-1),(D-1\right)} \end{array} \end{array}$$

$$=\int \ldots \int \xi_{D,D}\left|\frac{\partial \left(\xi_{1,1},\xi_{2,2},\ldots ,\xi_{\left(D-1),(D-1\right)}\right)}{\partial (K_{1},K_{2},\ldots ,K_{D-1})}\right|{\text{dK}}_{1}\ldots {\text{dK}}_{D-1}.$$

By replacing *ξ*_*k*.*k*_ by ${\tilde{\xi }}_{k,k}$ [see (8)] in the above definitions of *ROC*,*AUC* and the *VUS*, we obtain corresponding expressions for the linear classifier in (5). We denote the resulting ones as $\text{AUC}(\vec{n})$ and $\text{VUS}(\vec{n})$. In Additional file 3: Appendix 4, we derive these expressions for the case *D*=3.

### Computation of ***VUS***

As is evident from (9), the computation of *VUS* involves high dimensional integration. Given below is a brief description of the steps involved in the computation of *VUS*. For ease of exposition, we will denote *ξ*_*k*_=*ξ*_*k*,*k*_, *k*=1,…,*D*. First, we randomly generate the thresholds $\vec{K}=(K_{1},K_{2},\ldots ,K_{D-1})$ (see (9)) and compute the corresponding $\vec{\xi }={\left(\xi_{1},\xi_{2},\ldots ,\xi_{D}\right)}^{\prime }$ satisfying (7). Note that the $\vec{\xi }$ contributes to the integration in *VUS* only if all the *ξ*_*k*_’s are positive.

To find as many $\vec{\xi }$ values that contribute to the integration as possible, we use the *ant colony* optimization algorithm, where only the $\vec{K}$ values corresponding to the $\vec{\xi }$ values that contribute to the integration are retained. However, these are perturbed by a small noise and the resulting $\vec{K}$ values are used as seeds for the next iteration. Then, we use the genetic algorithm to obtain another $\vec{\xi }$ value located in a different region within (0,1)^*k*^, which also contributes to the integration. We use the *ant colony* algorithm and the genetic algorithm alternatively to eventually generate a dense set of $\vec{\xi }\;(\in {\left(0,1\right)}^{k})$ values that contribute to the integration. Note that the process is such that the newly generated $\vec{\xi }$ values are appended to all the previously generated $\vec{\xi }$ values.

Now, to compute the volume, *VUS*(*∞*), we use the *convhulln* function in the *qhullR*-package. Note that the *convhulln* function is designed to determine the convex hull of a set of *D*-dimensional points and thus compute the volume of the hull. In view of this, in order to compute the volume, *VUS*(*∞*), a base of $\vec{\xi }$ (this is same as the $\vec{\xi }$ vector, except that one of its components, e.g. the first component, is set to 0) is appended to the original $\vec{\xi }$. Since in each iteration the new $\vec{\xi }$ values are appended to the old $\vec{\xi }$ values from the previous iterations, and the *VUS* is concave, the computed *VUS* is supposed to increase in value with each iteration. We stop appending the new $\vec{\xi }$ values when |*VUS*_*o* *l* *d*_−*VUS*_*n* *e* *w*_|<0.001. When this criterion is satisfied, we obtain the value of *VUS*(*∞*). Similarly, the values of $\text{AUC}(\infty ),\text{AUC}(\vec{n}),\;\text{and}\;\text{VUS}(\vec{n})$ are calculated.

### Sample size determination using ***VUS*** or ***AUC***

Given a threshold *γ*, we determine the sample size *n* satisfying the following condition:

(10) $$\begin{array}{l} \text{VUS}(\infty )-\text{VUS}(\vec{n})<\gamma \end{array}$$

For the case *D*=2, we determine the sample size *n* satisfying the condition: $\text{AUC}(\infty )-\text{AUC}(\vec{n})<\gamma$. A simulation study for the case *D*=2 is carried out in Additional file 3: Appendix 5 to assess the performance of our sample size determination algorithm.

## Results

### Monte Carlo simulations

Before we illustrate the performance of our sample size determination method based on *AUC* or *VUS*, we present results from an extensive Monte Carlo simulation study conducted to verify the accuracy of the approximations for $\text{AUC}(\vec{n})$ and $\text{VUS}(\vec{n})$, respectively, and study their behavior as a function of *n* and other parameters. Here, we present the numerical assessments based on the *VUS* for the cases *D*=3 and 4, respectively. However, as mentioned above, the assessments based on the *AUC* for the case *D*=2 are given in Additional file 3: Appendix 5. Henceforth, we will set *n*_*k*_=*n* for all *k*=1,…,*D*, and we will use *n* instead of $\vec{n}$ to simplify notations.

When *D*=3, we consider the following simulation set up: For ${\vec{\theta }}_{1}={\left(\theta_{1,1},\ldots ,\theta_{1,m}\right)}^{\prime }$, let *θ*_1,*j*_∼*U*(0.4,0.49), *j*=1,…,*m*; for a specified scalar value *h*, let ${\vec{h}}_{1},{\vec{h}}_{2}$ be such that their components *h*_*i*,*j*_∼*U*(*h*−0.002,*h*+0.002), *i*=1,2; *j*=1,…,*m*; and let ${\vec{\theta }}_{2}={\vec{\theta }}_{1}-{\vec{h}}_{1}$, ${\vec{\theta }}_{3}={\vec{\theta }}_{2}-{\vec{h}}_{2}$. First, we generated a $\left({\vec{\theta }}_{1},{\vec{\theta }}_{2},{\vec{\theta }}_{3}\right)$ according to the above set up, and then generated the data vector $\vec{x}={\left(x_{1},\ldots ,x_{m}\right)}^{\prime }$ for each class. We then computed $\hat{\text{VUS}(\infty )}$ and $\hat{\text{VUS}(n)}$ following the computational methodology described earlier. For this $\left({\vec{\theta }}_{1},{\vec{\theta }}_{2},{\vec{\theta }}_{3}\right)$, we then drew twenty $\vec{x}$ data sets and calculated a Monte Carlo estimate, denoted by $\hat{\text{VUS}(n)}\text{MC}$. This process was repeated 20 times and an average value of $\hat{\text{VUS}(n)}\text{MC}$ was computed. These are given in Table 1. It is evident from Table 1 that the $\hat{\text{Bias}}=\hat{\text{VUS}(n)}\text{MC}-\hat{\text{VUS}(n)}$ is negligible in most cases, which validates the use of our approximation for *VUS*(*n*). Table 1 also gives similar results for the case *D*=4. Note that *VUS*(*∞*)=1/*D*! for a random classifier, which is the lower bound of *VUS*(*∞*) for any classifier.

**Table 1:** Performance of optimal and linear classifiers

			***D = 3***			
***h***	***m***	***n***	$\hat{\text{VUS}(\infty )}$	$\hat{\text{VUS}(n)}$	$\hat{\text{VUS}(n)\text{MC}}$	$\hat{\text{Bias}}$
0.02	50	50	0.3013	0.1772	0.1657	-0.0116
0.02	50	100	0.3015	0.1793	0.1742	-0.0052
0.02	100	50	0.3662	0.1807	0.1874	0.0067
0.02	100	100	0.366	0.1837	0.1974	0.0136
0.05	50	50	0.5469	0.2229	0.2442	0.0213
0.05	50	100	0.5467	0.2517	0.2845	0.0328
0.05	100	50	0.6988	0.2448	0.2912	0.0463
0.05	100	100	0.6987	0.2848	0.3377	0.0529
0.1	50	50	0.8686	0.4179	0.4675	0.0496
0.1	50	100	0.8687	0.4958	0.55	0.0542
0.1	100	50	0.9667	0.4776	0.5342	0.0566
0.1	100	100	0.9667	0.5692	0.6341	0.0649
			***D = 4***			
**h**	**m**	**n**	$\hat{\text{VUS}(\infty )}$	$\hat{\text{VUS}(n)}$	$\hat{\text{VUS}(n)\text{MC}}$	$\hat{\text{Bias}}$
0.02	50	50	0.1319	0.048	0.0462	-0.0018
0.02	50	100	0.1318	0.05	0.0512	0.0013
0.02	100	50	0.1892	0.0503	0.057	0.0068
0.02	100	100	0.189	0.0531	0.0614	0.0082
0.05	50	50	0.3891	0.0893	0.0923	0.003
0.05	50	100	0.3893	0.1175	0.1144	-0.0032
0.05	100	50	0.5832	0.1092	0.1127	0.0034
0.05	100	100	0.5831	0.1458	0.1285	-0.0174
0.1	50	50	0.8376	0.2933	0.2705	-0.0228
0.1	50	100	0.8378	0.4059	0.3517	-0.0542
0.1	100	50	0.9623	0.3653	0.3119	-0.0534
0.1	100	100	0.9626	0.4962	0.4085	-0.0877

Here, *D *= 3 and 4, ${\vec{\theta }}_{1}={\left(\theta_{1,1},\ldots ,\theta_{1,m}\right)}^{\prime }$, let *θ*_1,*j*_∼*U*(0.4,0.49),*j*=1,…,*m*; for a specified scalar value *h*, let ${\vec{h}}_{1},{\vec{h}}_{2},{\vec{h}}_{3}$ be such that their components *h*_*i*,*j*_∼*U* (*h*−0.002,*h*+0.002),*j*=1,…,*m*; and let ${\vec{\theta }}_{i+1}={\vec{\theta }}_{i}-{\vec{h}}_{i},i=1,2,3$; *n* is the sample size for each class; *m* is the number of independent SNPs, *α*=0.01 is the significant level for Wald tests; and *ρ*=1 is the percentage of the significant SNPs.

Next, we determine the smallest *n* such that $f(n)=\hat{\text{VUS}(\infty )}-\hat{\text{VUS}(n)}-\gamma <0$, for a pre-specified *γ* value. We use the following algorithm to determine such an *n*: (i) Let *n*=*n*_*S*_ and *n*_*L*_ such that *f*(*n*_*S*_)>0 and *f*(*n*_*L*_)<0, and set *n*_*M*_=[(*n*_*S*_+*n*_*L*_)/2]. The algorithm begins by selecting a small *n*_*S*_ and a large *n*_*L*_; (ii) If *f*(*n*_*M*_)*f*(*n*_*S*_)<0, then reset *n*_*L*_=*n*_*M*_; or else, reset *n*_*S*_=*n*_*M*_. In either case, return to step (i), unless *n*_*L*_−*n*_*S*_≤1, in which case, the smallest sample *n*=*n*_*L*_; (iii) Use the smallest (total) sample of size *D*×*n*_*L*_, with *n*=*n*_*L*_ from each class, *C*_1_,…,*C*_*D*_. We implemented this algorithm for each value of *h*, *m* and significance level *α* for the Wald test; see discussion below (5). For the cases *D*=2 and *D*=3, respectively, Table 2 displays the determined sample sizes for *γ*=0.01 and each combination of parameter values. From Table 2, it is evident that the required sample size reduces as *h* increases, as expected. Hence, *f*(*n*)<0 for smaller sample sizes, as shown in Table 2. However, the effect of *m* on the determined sample sizes is less clear. When *h* is large, say *h*≥0.1, then the required sample size reduces as *m* becomes large. Whereas, when *h* is small, say *h*=0.05, the reverse is true as *m* becomes large.

**Table 2:** **Sample size determination: here, *D = 3 * and *4*, and *n* is the sample size for each class satisfying:**$\hat{\text{VUS}(\infty )}-\hat{\text{VUS}(n)}<\gamma \;(=0.01)$

		***n***			
***D***	***h***	***m = 30***	***m = 50***	***m = 100***	***m = 200***
3	0.05	1957	2040	2091	2040
3	0.1	489	475	412	288
3	0.15	189	161	105	69
4	0.05	1923	2051	2137	2122
4	0.1	490	476	417	297

Here, ${\vec{\theta }}_{1}={\left(\theta_{1,1},\ldots ,\theta_{1,m}\right)}^{\prime }$, let *θ*_1,*j*_∼*U*(0.4,0.49),*j*=1,…,*m*; for a specified scalar value *h*, let ${\vec{h}}_{1},{\vec{h}}_{2},{\vec{h}}_{3}$ be such that their components *h*_*i*,*j*_∼*U*(*h*−0.002,*h*+0.002), *j*=1,…,*m*; and let ${\vec{\theta }}_{i+1}={\vec{\theta }}_{i}-{\vec{h}}_{i},i=1,2,3$; *m* is the number of independent SNPs, *α*=0.01 is the significant level for Wald tests; and *ρ*=1 is the percentage of the significant SNPs.

#### Application to the HapMap data

The aim of the International HapMap Project is to develop a haplotype map of the human genome, which will describe the common patterns of human DNA sequence variation.

The HapMap data (Phase III) consists of eleven populations with about *p*=1.2×10^6^ SNPs. Here, we consider the following nine populations in order to illustrate our sample size determination algorithm: ASW—African ancestry in Southwest USA with 87 subjects; CEU—Utah residents with Northern and Western European ancestry from CEPH collection with 167 subjects; CHB—the Han Chinese individuals from Beijing with 137 subjects; CHD—Chinese in Metropolitan Denver, Colorado with 109 subjects; GIH—Gujarati Indians in Houston, Texas with 101 subjects; JPT—the Japanese individuals from Tokyo with 113 subjects; MEX—Mexican ancestry in Los Angeles, California with 86 subjects; TSI—Toscans in Italy (TSI) with 102 subjects; and YRI—Yoruba in Ibadan, Nigeria with 203 subjects. With these, we created four sample size determination studies, of which the first three involve three populations (*D*=3), and the last study involves four populations (*D*=4). More specifically, we conducted our sample size determination studies with the following population groupings: (I) (CEU, GIH, MEX); (II) (ASW, TSI, YRI); (III) (CHB, JPT, CHD); and (IV) (CHB, JPT, CHD, GIH).

Based on all the available subjects, we extracted pair-wise independent SNPs using the following steps. Suppose *L* is a set of SNPs, then: (I) form a set *S* with one SNP from *L* and update *S* after the next step; (II) from the remaining SNPs in *L*, choose one SNP that is independent of every SNP in *S* using Kendall’s *τ* coefficient as a test statistic to test pair-wise independence, and then add this new SNP to *S*. Here, we concluded independence if the Kendall’s *τ*-value <0.05; (III) Repeat (II) until each remaining SNP in *L* is correlated with at least one SNP in *S*. This procedure yielded a set *S* with *m*=92 pair-wise independent SNPs, and with these we built our linear classifier.

**Figure 1:**
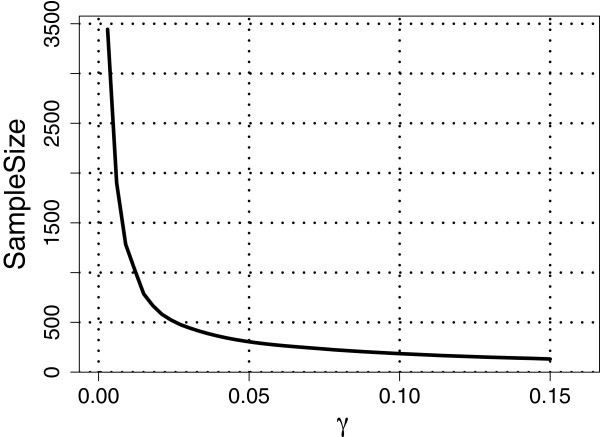
**Total sample sizes needed for classification to *well-separated* HapMap populations CEU, GIH, and MEX.** For the linear classifier based on the SNP data from the three populations, the estimated learning curve gives the required total sample size for different values of the threshold, *γ*, satisfying $\hat{\text{VUS}(\infty )}-\hat{\text{VUS}(n)}<\gamma$. Here, *ρ* = 1, *α* = 0.1, *m* = 92, and $\hat{\text{VUS}(\infty )}=0.9046$.

**Figure 2:**
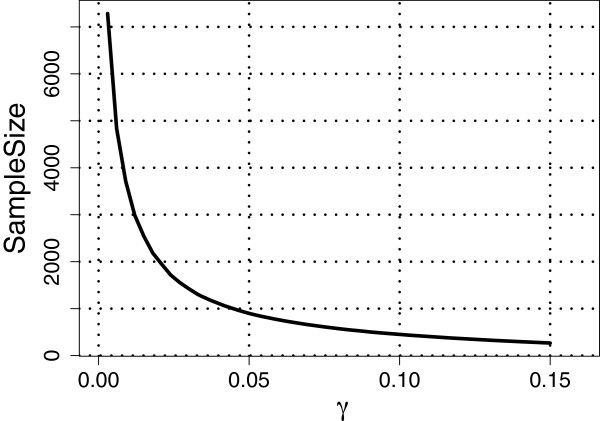
**Total sample sizes needed for classification to *moderately-separated* HapMap populations ASW, TSI, and YRI.** For the linear classifier based on the SNP data from the three populations, the estimated learning curve gives the required total sample size for different values of the threshold, *γ*, satisfying $\hat{\text{VUS}(\infty )}-\hat{\text{VUS}(n)}<\gamma$. Here, *ρ *= 1, *α* = 0.1, *m* = 92, and $\hat{\text{VUS}(\infty )}=0.7557$.

**Figure 3:**
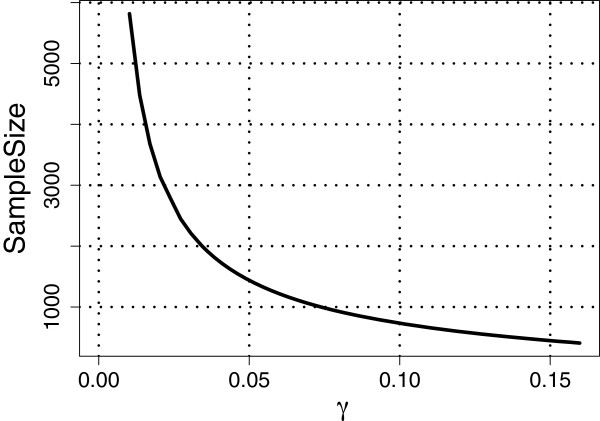
**Total sample sizes needed for classification to *poorly-separated* HapMap populations CHB, JTP, and CHD.** For the linear classifier based on the SNP data from the three populations, the estimated learning curve gives the required total sample size for different values of the threshold, *γ*, satisfying $\hat{\text{VUS}(\infty )}-\hat{\text{VUS}(n)}<\gamma$. Here, *ρ *= 1, *α* = 0.1, *m* = 92, and $\hat{\text{VUS}(\infty )}=0.6178$.

**Figure 4:**
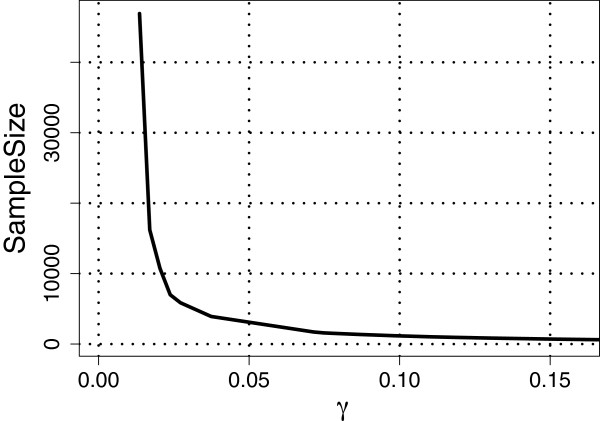
**Total sample sizes needed for classification to majority *poorly-separated* HapMap populations CHB, JTP, CHD and GIH.** For the linear classifier based on the SNP data from the three populations, the estimated learning curve gives the required total sample size for different values of the threshold, *γ*, satisfying $\hat{\text{VUS}(\infty )}-\hat{\text{VUS}(n)}<\gamma$. Here, *ρ *= 1, *α* = 0.1, *m* = 92, and $\hat{\text{VUS}(\infty )}=0.5580$.

Next, we set *ρ*=1 so that *m*=*l*=92; see Assumption 3 under the Methods section. Recall that $\vec{\theta_{k}}={\left(\theta_{k,1},\ldots ,\theta_{k,l}\right)}^{\prime }$ for *k*=1,…,*D*. For the cases *D*=3 and *D*=4 considered in studies (I) to (IV) above, we estimated $\vec{\theta_{k}}$ using the maximum likelihood (ML) estimates obtained based on all the available subjects belonging to the respective populations. We then substituted these ML estimates into the corresponding expressions for $\hat{\text{VUS}(\infty )}$ and $\hat{\text{VUS}(n)}$, respectively. Figures 1, 2 and 3 show plots of required sample sizes for a continuum of threshold values *γ* for the case *D*=3 considered in studies (I) to (III), respectively, and Figure 4 plots the same for *D*=4 considered in study (IV). From these figures, the required total sample size can be determined approximately for each pre-specified *γ* value.

For example, if we set *γ*=0.10 (i.e., $\hat{\text{VUS}(\infty )}-\hat{\text{VU}}S(n)<0.10$), then in the three population (CEU,GIH, MEX) case, the $\hat{\text{VUS}(\infty )}=0.9046$ and about 62 observations are required for each class with a total sample size of 186, whereas in the three population (ASW, TSI, YRI) case, the $\hat{\text{VUS}(\infty )}=0.7557$ and about 150 observations are required for each class with a total sample size of 450. Note that, for *γ*=0.10, in study (I) the required sample sizes for each population is less than what is currently available, whereas in study (II), we would need 63 and 48 more observations for the populations ASW and TSI, respectively. For the three population (CHB, JPT and CHD) case, if we set *γ*=0.10 then the $\hat{\text{VUS}(\infty )}=0.6178$ and about 244 observations are required for each class with a total sample size of 732. Clearly, for study (III) at least 100 more observations are needed for each population (CHB, JPT and CHD) when *γ*=0.10. Finally, for the four population (CHB, JPT, CHD, GIH) case, setting *γ*=0.10 yields that the $\hat{\text{VUS}(\infty )}=0.5580$ and about 279 samples are required for each class with a total sample of 1,116. Once again, at least 150 more observations are needed for each of the four populations when *γ*=0.10.

The results from the four HapMap studies suggest that the $\hat{\text{VUS}(\infty )}$ value is large and the required total sample size is small when the populations are *well-separated* [as in study (I)]. Whereas, when the populations are *moderately-separated* [as in study (II), where the populations ASW and YRI may be similar], the $\hat{\text{VUS}(\infty )}$ value reduces and the required total sample size increases moderately. When the populations are *poorly-separated* [as in study (III), where all the three populations may be similar], the $\hat{\text{VUS}(\infty )}$ value reduces even further and there is a substantial increase in the required total sample size. Finally, in the four population study, where three of the populations are *poorly-separated*, once again we see a further reduction in the $\hat{\text{VUS}(\infty )}$ value and a corresponding increase in the required total sample size. Although not reported here, we also considered other *well-/moderate-/poorly- separated* cases with the HapMap data and observed similar results as the ones reported here.

It is well known in the classification literature that the performance of a classifier depends on how well separated the classes are. Similarly, the studies above involving the HapMap data show that the performance of our sample size determination methodology also depends on the extent of separation between populations. While our methodology provides a formal way of determining an approximate total sample size for each specified value of *γ*, it is clear from the HapMap data analysis that it is not possible to propose a universal *γ* value. Nevertheless, if the classes are *well-separated* or *moderately-separated*, then we believe that *γ*=0.10 may be a good choice for many frequently encountered data sets in classification problems.

## Discussion

We have built an optimal Bayes classifier and a linear classifier based on coded SNP data from two or more classes. For these classifiers, we have considered the two commonly used scalar performance measures, the Area Under the *ROC* curve (*AUC*) and the Volume Under the *ROC* hyper-Surface (*VUS*), which allow classifiers to be compared independent of discrimination values. We have illustrated the performance of a sample size determination methodology, which selects the smallest total sample size *n* such that the criterion $\hat{\text{VUS}(\infty )}-\hat{\text{VUS}(n)}<\gamma$ is satisfied. While the approximations to the *VUS* (or *AUC*) obtained here provide the necessary theoretical justification, the simulations and the HapMap data analysis presented here illustrate the practical value of our sample size determination method.

The fact that the *HapMap* contains data on multiple populations belonging to similar or dissimilar geographical locations enabled us to test the performance of our sample size determination method on three different multi-class scenarios involving *well-separated*, *moderately-separated*, and *poorly-separated* populations. We have shown that the the extent of separation between the populations and the choice of threshold value affect the total sample size required to satisfy the criterion. With regard to the choice of the threshold value *γ* in other practical contexts, we recommend that the user take into consideration the cost of obtaining more samples and choose an appropriate value of *γ* that gives an acceptable precision. In other words, if the cost of sampling is affordable then the user may want to sample more to achieve a higher precision (lower *γ* value) using our classifier; otherwise, the user has to settle for a higher *γ* value that makes use of all the available samples. We also infer from our HapMap data analysis that a value of $\hat{\text{VUS}(\infty )}>0.80$ may indicate the extent of separation between the classes. Thus, the value of $\hat{\text{VUS}(\infty )}$ could also give some prior guidance on the choice of *γ* values, especially in instances where the cost of sampling is a serious concern.

## Conclusion

In summary, for multiple classes, we have developed an asymptotic methodology based on *AUC* or *VUS* to estimate the learning curve of SNP classifiers. It is shown that the required total sample size can be obtained from the estimated learning curve for each pre-specified threshold value. In classification problems, sample size determination is important due to cost considerations. This methodology will help scientists determine if a sample at hand is adequate or more observations are necessary to achieve a pre-specified accuracy, and thus help users strike an optimal balance between precision and cost.

## Competing interests

The authors declare that they have no competing interests.

## Authors’ contributions

XL developed and implemented the proposed model, performed simulation and application, and drafted the manuscript. TNS participated in model development and helped manuscript preparation. YW participated in HapMap data analysis. All authors read and approved the final manuscript.

## Supplementary Material

Additional file 1 Manual of R package “SampleSizeSN”. Click here for file

Additional file 2 R package “SampleSizeSNP” in ZIP file. Click here for file

Additional file 3 Appendix 1–5. Click here for file
