# Supplementary material for: Determination of sample size for a multi-class classifier based on single-nucleotide polymorphisms: a volume under the surface approach
Source: BMC Bioinformatics. 2014 Jun 14;15:190. doi: 10.1186/1471-2105-15-190 (PMC4071155; doi:10.1186/1471-2105-15-190)
Supplement: Additional file 1 — Manual of R package “SampleSizeSN”. [file 1471-2105-15-190-S1.pdf]

Package ‘SampleSizeSNP’

January 21, 2014

Type Package

Title Sample size determination for classifier based on SNP.

Version 1.0

Date 2012-11-07

Author Xinyu Liu <xinyu81@uga.edu>

Maintainer Xinyu Liu <xinyu81@uga.edu>

Depends mvtnorm, geometry,DEoptim,e1071,ROCR,pROC

Description This package implements a version of sample size determination for SNP classifier. It can be based on the Probability of Correct Classification (PCC) or Area Under Curve (AUC).

License GPL-2

R topics documented:

AUC.inf 2
AUC.n 3
AUC.Simulation 4
Compute.MAF 5
Find.SS.AUC 6
Find.SS.PCC 7
Find.SS.VUS 9
Generate.Theta 10
Hapmap51 11
Hapmap92 12
HS348 13
PCC.inf.optimal 14
PCC.n.optimal 15
ROC.4Figures 16
ROC.infinity.plot 18
ROC.n.plot 19
VUS.inf 20
VUS.n 21

Index **23**

`AUC.inf` *Compute the AUC under theoretical maximum PCC.*

Description

Compute the AUC under theoretical maximum PCC.

Usage

```
AUC.inf(theta1, theta2, rho)
```

Arguments

`theta1`	The MAF of case groups.
`theta2`	The MAF of control groups.
`rho`	The percentage of SNP's with effect on case/control, default = 1.

Value

The AUC value.

Author(s)

Xinyu Liu

References

Liu, X., et al. Sample size determination for classifiers based on single-nucleotide polymorphisms. Biostatistics (2012), 13:2, 217-227.

Examples

```
m <- 10 # number of SNPs
h <- 0.1 # the difference between two MAFs
theta1 <- rep(0.3,10)
theta2 <- theta1 - 0.1
AUC.inf(theta1,theta2,rho=1)
#########################
data(Hapmap51)
AUC.inf(Hapmap51$theta1,Hapmap51$theta2,rho=1)
```

AUC.n *AUC under linear classifier.*

Description

The AUC of the linear classifier.

Usage

```
AUC.n(n1, n2, theta1, theta2, alpha, rho)
```

Arguments

n1	The number of observations in case group.
n2	The number of observation in control groups.
theta1	The MAF for case groups.
theta2	The MAF for control groups.
alpha	The significant level to select the SNPs, default = 0.1.
rho	The percentage of SNPs with effect on case/control, default = 1.

Value

The AUC number based on the linear classifier.

Author(s)

Xinyu Liu

References

Liu, X., et al. Sample size determination for classifiers based on single-nucleotide polymorphisms. Biostatistics (2012), 13:2, 217-227.

Examples

```
m <- 10 # number of SNPs
h <- 0.1 # the difference between two MAFs
n1 <- 50
n2 <- 50
theta1 <- rep(0.3,m)
theta2 <- theta1 - 0.1
alpha <- 0.01
rho=1
AUC.n(n1, n2, theta1, theta2,alpha,rho)
#########################
data(Hapmap51)
AUC.n(n1, n2, Hapmap51$theta1, Hapmap51$theta2,alpha,rho)
```

AUC.Simulation *Compute the values for PCC(inf), PCC(n)_f, PCC(n)_MC, and PCC(n)_SVM.*

Description

Compute the values for PCC(inf), PCC(n)_f, PCC(n)_MC, and PCC(n)_SVM.

Usage

```
AUC.Simulation(h_list,m_list,alpha_list,n_list,low,high,bin_num,pi_1,rho,kkk)
```

Arguments

h_list	The list of h values.
m_list	The list of m values.
alpha_list	The list of alpha values.
n_list	The list of n values.
low	Lower bound of threshold K's.
high	Upper bound of threshold K's.
bin_num	The number of bins for threshold.
pi_1	The percentage of group 1.
rho	The percentage of significant SNP's.
kkk	The times of repeat in the simulation.

Details

Compute the values for PCC(inf), PCC(n)_f, PCC(n)_MC, and PCC(n)_SVM.

Value

The sample size.

Author(s)

Xinyu Liu

References

Liu, X., et al. Sample size determination for classifiers based on single-nucleotide polymorphisms. Biostatistics (2012), 13:2, 217-227.

Examples

```
theta1 <-  0.3
alpha = 0.01
rho = 1
low=-10
high=200
bin_num=5000
pi_1=pi_2=0.5
kkk=200
h_list = c(0.01,0.05,0.1,0.2)
m_list = c(10,50,200)
alpha_list = c(0.01)
n_list = c(30,100,200)
#AUC.Simulation(h_list,m_list,alpha_list,n_list,low,high,bin_num,pi_1,rho,kkk)
```

Compute.MAF *Compute the MAF given the sample.*

Description

To determine the sample size, the minor allele frequency (MAF) of case and control groups are needed; if they are not available, then the intial sample of the SNP data with case/control labels are required, and the MAF of two groups will be estimated by MLE.

Usage

```
Compute.MAF(data, label)
```

Arguments

data	The SNP data set, which is encoded by the number of minor allele.
label	The label of case/control, please be note that the case group is labeled by higher number and corresponds to theta1, and the control group is labeled by lower number and corresponds to theta2.

Value

MLE of theta1 and theta2.

Author(s)

Xinyu Liu

References

Liu, X. et al. (2012) Sample size determination for classifiers based on single-nucleotide polymorphisms. Biostatistics, 13:2, 217-277.

Examples

```
data(Hapmap51)
Compute.MAF(Hapmap51$data,Hapmap51$labels)
```

`Find.SS.AUC` *Determine the sample size based on the AUC.*

Description

Find the sample size such that AUC(infinity) - AUC(n) < diff

Usage

```
Find.SS.AUC(RequestDifference,diff,pi_1,theta1,theta2,alpha,rho)
```

Arguments

`RequestDifference`	If TRUE, then it will find the sample size such that AUC(inf)-AUC(n) < diff, if FALSE, then it will find that sample size such that 1 - AUC(n) < diff. Please see the details and examples.
`diff`	The threshold of AUC(infinity) - AUC(n).
`pi_1`	The percentage of case group in sample data, so n1/n2 = pi_1/(1-pi_1)
`theta1`	The MAF of case group.
`theta2`	The MAF of case group.
`alpha`	The significant level of selecting the SNPs by Wald test, default = 0.01.
`rho`	The percentage of SNP with the effect on case/control, default = 1.

Details

This function determines the sample size needed according to AUC, it is devided into two parts, the first part as stated in the paper, is to find the sample size, n, such that AUC(infinity)-AUC(n) <diff. The second part ignores the AUC(infinity), only consider the AUC(n), and find the sample size, n, such that 1 - AUC(n) < diff, or 1-diff < AUC(n). But before that, please be note that the AUC(n) can not exceed AUC(infinity), and can not be lower than AUC(n), so remember to check the range of AUC(n) before setting the threshold diff. Please see the examples for more details.

Value

The sample size.

Author(s)

Xinyu Liu

References

Liu, X., et al. Sample size determination for classifiers based on single-nucleotide polymorphisms. Biostatistics (2012), 13:2, 217-227.

Examples

```
data(Hapmap51)
### first find the theoretical maximum value of AUC, AUC(infinity).
AUC.inf(Hapmap51$theta1,Hapmap51$theta2,rho=1)
### second find the lowest value of AUC.
AUC.n(1,1,Hapmap51$theta1,Hapmap51$theta2,alpha=0.1,rho=1)
### so the AUC(n) should between these two values.

### Or you can make the plot of AUC(n) and AUC(inf)
res <- matrix(NA, nrow=1,ncol=100)
for (i in 1:100)
{
    res[1,i] <- AUC.n(i*5,i*5,Hapmap51$theta1,Hapmap51$theta2,alpha=0.1,rho=1)
}
plot(10*seq(100),res,type="l",lwd=3,xlab="Sample Size",ylab="AUC") #please be note that the sample size is 2
abline(h = AUC.inf(Hapmap51$theta1,Hapmap51$theta2,rho=1),lwd=3)


### Then you can find the sample size according to the threshold, diff.
### if you want to find the sample size such that AUC(inf)-AUC(n) < diff, then you can use
Find.SS.AUC(RequestDifference=TRUE, diff=0.01,pi_1=0.5,theta1=Hapmap51$theta1,theta2=Hapmap51$theta2,alpha=

### or you just want to control AUC(n) and ignore AUC(infinity), then the formula becomes
### 1- AUC(n) < diff, or AUC(n) > 1 - diff, you can use
Find.SS.AUC(RequestDifference=FALSE, diff=0.3,pi_1=0.5,theta1=Hapmap51$theta1,theta2=Hapmap51$theta2,alpha=
```

`Find.SS.PCC` *Compute the sample size based on PCC.*

Description

Find the sample size such that PCC(infinity) - PCC(n) < diff

Usage

```
Find.SS.PCC(RequestDifference,diff,pi_1,theta1,theta2,alpha,rho)
```

Arguments

`RequestDifference`	If TRUE, then it will find the sample size such that PCC(inf)-PCC(n) < diff, if FALSE, then it will find that sample size such that 1 - PCC(n) < diff. Please see the details and examples.
`diff`	The thresold for the difference of two PCCs.
`pi_1`	The percentage of the case group, so n1/n2 = pi_1/(1-pi_1).
`theta1`	The MAF of case group.
`theta2`	The MAF of control group.
`alpha`	The significant level to choose the SNPs, default = 0.1.
`rho`	The percentage of SNP with effect on case/control, default = 1.

Details

This function determines the sample size needed according to PCC, it is devided into two parts, the first part as stated in the paper, is to find the sample size, n, such that PCC(infinity)-PCC(n) < diff. The second part ignores the PCC(infinity), only consider the PCC(n), and find the sample size, n, such that 1 - PCC(n) < diff, or 1-diff < PCC(n). But before that, please be note that the PCC(n) can not exceed PCC(infinity), and can not be lower than PCC(n), so remember to check the range of PCC(n) before setting the threshold diff. Please see the examples for more details.

Value

The sample size.

Author(s)

Xinyu Liu

References

Liu, X., et al. Sample size determination for classifiers based on single-nucleotide polymorphisms. Biostatistics (2012), 13:2, 217-227.

Examples

```
data(Hapmap51)
### first find the theoretical maximum value of PCC, PCC(infinity).
PCC.inf.optimal(Hapmap51$theta1,Hapmap51$theta2,pi_1=0.5,rho=1)
### second find the lowest value of PCC.
PCC.n.optimal(1,Hapmap51$theta1,Hapmap51$theta2,pi_1=0.5,alpha=0.1,rho=1)
### so the PCC(n) should between these two values.

### Or you can make the plot of AUC(n) and AUC(inf)
res <- matrix(NA, nrow=1,ncol=100)
for (i in 1:100)
{
   res[1,i] <- PCC.n.optimal(i*5,Hapmap51$theta1,Hapmap51$theta2,pi_1=0.5,alpha=0.1,rho=1)
}
plot(10*seq(100),res,type="l",lwd=3,xlab="Sample Size",ylab="PCC") #please be note that the sample size is 2
abline(h = PCC.inf.optimal(Hapmap51$theta1,Hapmap51$theta2,pi_1=0.5,rho=1),lwd=3)


### Then you can find the sample size according to the threshold, diff.
### if you want to find the sample size such that PCC(inf)-PCC(n) < diff, then you can use
Find.SS.PCC(RequestDifference=TRUE, diff=0.01,pi_1=0.5,theta1=Hapmap51$theta1,theta2=Hapmap51$theta2,alpha=

### or you just want to control PCC(n) and ignore PCC(infinity), then the formula becomes
### 1- PCC(n) < diff, or PCC(n) > 1 - diff, you can use
Find.SS.PCC(RequestDifference=FALSE, diff=0.3,pi_1=0.5,theta1=Hapmap51$theta1,theta2=Hapmap51$theta2,alpha=
```

`Find.SS.VUS` *Determine the sample size based on the VUS.*

Description

Find the sample size such that VUS(infinity) - VUS(n) < diff

Usage

```
Find.SS.VUS(diff,C,Theta,alpha,m,kk,rho,abserr)
```

Arguments

`diff`	The threshold of VUS(infinity) - VUS(n).
`C`	Number of groups.
`Theta`	The MAF matrix, with m rows and C columns.
`alpha`	The significant level of selecting the SNPs by Wald test.
`m`	Number of SNP's.
`kk`	The number of random values generated to compute the integration by qhull method.
`rho`	The percentage of SNP with the effect on case/control, default = 1.
`abserr`	The cutoff of the error.

Details

This function determines the sample size needed according to VUS, it is devided into two parts, the first part as stated in the paper, is to find the sample size, n, such that VUS(infinity)-VUS(n) <diff. The second part ignores the VUS(infinity), only consider the VUS(n), and find the sample size, n, such that 1 - VUS(n) < diff, or 1-diff < VUS(n). But before that, please be note that the VUS(n) can not exceed VUS(infinity), and can not be lower than VUS(n), so remember to check the range of VUS(n) before setting the threshold diff. Please see the examples for more details.

Value

The sample size.

Author(s)

Xinyu Liu

References

Liu, X., et al. Sample size determination for classifiers based on single-nucleotide polymorphisms. Biostatistics (2012), 13:2, 217-227.

Examples

```
rho = 1
alpha=0.01
C=3
kk=100
kkk=200
rr=10
U1=0.4
U2=0.49
Di=0.002
abserr = 0.001
ms = 1
diff <- 0.01
theta_1_list = c(0.3)
h_list = c(0.05,0.1,0.15)
m_list = c(30,50,100,200)
alpha_list = c(0.01)
rho=1
#Results_Tab3 = matrix(ncol=4,nrow=length(theta_1_list)*length(h_list)*length(m_list)*length(alpha_list))
#colnames(Results_Tab3) <- c("h","m","alpha","Size")
#mm = 1
#for (theta1 in theta_1_list)
#{
#    for (h in h_list)
#    {
#        for (m in m_list)
#        {
#            for (alpha in alpha_list)
#            {
#                ll <- m
#                Theta <- Generate.Theta(C,m,U1,U2,h,Di,rho)$Theta
#                SampleS <- Find.SS.VUS(diff,C,Theta,alpha,m,kk,rho,abserr)
#                Results2 <- c(h,m,alpha,SampleS)
#                print(Results2)
#                Results_Tab3[mm,] <- Results2
#                mm = mm + 1
#            }
#        }
#    }
#}
```

`Generate.Theta` *Gererate matrix of MAF randomly.*

Description

This function produces the MAF matrix randomly, which is m*C, that is, each column corresponds to the MAF of m SNP's for one class. In this program, given SNPi, theta_i1 ~ U(U1,U2), h1,h2,...,h_(C-1) ~ U(h-Di,h+Di), theta_i2 = theta_i1 - h1,theta_i3 = theta_i2 - h2, and so on.

If rho < 1, then for each SNP (C groups) there is rho percentage that theta_i1,...,theta_iC ~ U(0.1,0.4)

Usage

```
Generate.Theta(C,m,U1,U2,h,Di,rho)
```

Arguments

C	Number of groups.
m	Number of SNP's.
U1	theta_i1 ~ U(U1,U2).
U2	theta_i1 ~ U(U1,U2).
h	h1,h2,...,h_(C-1) ~ U(h-Di,h+Di).
Di	h1,h2,...,h_(C-1) ~ U(h-Di,h+Di).
rho	The percentage of SNP's with effect on case/control, default = 1.

Value

$Theta: MAF matrix, with m rows and C columns. $significant_index : if 0, then theta_i1,...,theta_iC ~ U(0.1,0.4), if 1, then theta_i1 ~ U(U1,U2), h1,h2,...,h_(C-1) ~ U(h-Di,h+Di), theta_i2 = theta_i1 - h1,theta_i3 = theta_i2 - h2, and so on.

Author(s)

Xinyu Liu

References

Liu, X., et al. Sample size determination for classifiers based on single-nucleotide polymorphisms. Biostatistics (2012), 13:2, 217-227.

Examples

```
rho = 1
C=3
U1=0.4
U2=0.49
Di=0.002
h = 0.1
m = 50
Theta <- Generate.Theta(C,m,U1,U2,h,Di,rho)$Theta
Theta
```

Hapmap51 *The 51 selected encoded SNPs*

Description

The class is made of three elements, the first one "Hapmap51$data" is the SNPs data, it is from the encoded Hapmap data, and the 51 SNPs are selected by the "independent criteria" which is described by the paper below. The second one "Hapmap51$labels" are the labels of the case/control. "Hapmap51$theta1" and "Hapmap51$theta2" are MAF of case and contorl groups, respectively, which are estimated by maximum likelihood method.

Usage

```
Hapmap51
```

References

Liu, X., et al. Sample size determination for classifiers based on single-nucleotide polymorphisms. Biostatistics (2012), 13:2, 217-227.

Examples

```
data(Hapmap51)
dim(Hapmap51$data)
Hapmap51$data[1:10,1:10]
Hapmap51$labels
Hapmap51$theta1
Hapmap51$theta2
```

Hapmap92 *The 92 selected encoded SNPs*

Description

The class is made of three elements, the first one "Hapmap92$data" is the SNPs data, it is from the encoded Hapmap data, and the 92 SNPs are selected by the "independent criteria" which is described by the paper below. The second one "Hapmap51$labels" are the labels of the population. "Hapmap92$Theta" are MAF matrix, which are estimated by maximum likelihood method. The labels and corresponding number:

0 – African ancestry in Southwest USA (ASW, 87) 1 – Utah residents with Northern and Western European ancestry from the CEPH collection (CEU, 165) 2 – Han Chinese in Beijing, China (CHB, 137) 3 – Chinese in Metropolitan Denver, Colorado (CHD, 109) 4 – Gujarati Indians in Houston, Texas (GIH, 101) 5 – Japanese in Tokyo, Japan (JPT, 113) 6 – Luhya in Webuye, Kenya (LWK, 110) 7 – Mexican ancestry in Los Angeles, California (MEX, 86) 8 – Maasai in Kinyawa, Kenya (MKK, 184) 9 – Toscani in Italia (TSI, 102) 10 – Yoruba in Ibadan, Nigeria (YRI, 203)

Usage

```
Hapmap92
```

References

Liu, X., et al. Sample size determination for classifiers based on single-nucleotide polymorphisms. Biostatistics (2012), 13:2, 217-227.

Examples

```
data(Hapmap92)
dim(Hapmap92$data)
Hapmap92$data[1:10,1:10]
Hapmap92$labels[1:10]
Hapmap92$Theta[1:5,]
Theta3 <- Hapmap92$Theta[,c(3,4,6)]
rho = 1
```

```
abserr = 0.001
kk = 100
ll = m = 92
C <- 3
alpha <- 0.1
abserr = 0.001
n <- 100
N <- matrix(n,nrow=C,ncol=1)
#VUS.inf(C,ll,Theta3,kk,abserr) #0.6177627
#VUS.n(C,Theta3,alpha,N,m,rho,kk,abserr)  #0.4245822
```

HS348 *The 348 selected encoded SNPs from Heterogeneous Stock Mice Data*

Description

The class is made of five elements, the first one "data" is the SNPs data, it is from the encoded Heterogeneous Stock Mice data, and the 348 SNPs are selected by the "independent criteria" which is described by the paper below. "Anx_label_C3" is the categorical label of Anxiety according to its distribution, the cutoffs are 0.1 and 0.6. "MAF.Anx.C3" is the MAF according to the label "Anx_label_C3". Similarly, "ObeBMI_label_C4" are the categorical labels of Obesity BMI, the cutoffs are the four quantiles of Obesity BMI.

Usage

```
HS348
```

References

Liu, X., et al. Sample size determination for classifiers based on single-nucleotide polymorphisms. Biostatistics (2012), 13:2, 217-227.

Examples

```
data(HS348)
dim(HS348$data)
HS348$data[1:10,1:10]
rho = 1
abserr = 0.01
kk = 100
ll = m = 348
C <- 3
alpha <- 0.01
n <- 100
N <- matrix(n,nrow=C,ncol=1)
#Theta <- HS348$MAF.Anx.C3
#VUS.inf(C,ll,Theta,kk,abserr)  #0.6921208
#VUS.n(C,Theta,alpha,N,m,rho,kk,abserr)  #0.2467791
#Theta <- HS348$ObeBMI_MAF_C4
```

PCC.inf.optimal	*The theoretical maximum value of PCC.*

Description

The theoretical maximum value of PCC, which is computed from the assumptions and Bayesian formula.

Usage

```
PCC.inf.optimal(theta1, theta2, pi_1, rho)
```

Arguments

theta1	The MAF for case group.
theta2	The MAF for control group.
pi_1	The percentage of case group.
rho	The percentage of SNP with effect on case/control, default = 1.

Value

The PCC(infinity) value.

Author(s)

Xinyu Liu

References

Liu, X., et al. Sample size determination for classifiers based on single-nucleotide polymorphisms. Biostatistics (2012), 13:2, 217-227.

Examples

```
m <- 10
h <- 0.1
theta1 <- rep(0.3,m)
theta2 <- theta1 - h
pi_1 <- 0.5
PCC.inf.optimal(theta1, theta2, pi_1,rho=1)
#########################################################
data(Hapmap51)
PCC.inf.optimal(theta1=Hapmap51$theta1, theta2=Hapmap51$theta2, pi_1,rho=1)
```

PCC.n.optimal *The PCC under the linear classifier.*

Description

PCC(n)

Usage

```
PCC.n.optimal(n1, theta1, theta2, pi_1, alpha, rho)
```

Arguments

n1	The number of case group.
theta1	The MAF of case group.
theta2	The MAF of control group.
pi_1	The percentage of case group.
alpha	The significant level to select SNP, default = 0.1.
rho	The percentage of SNP with effect on case/control, default = 1.

Value

PCC(n)

Author(s)

Xinyu Liu

References

Liu, X., et al. Sample size determination for classifiers based on single-nucleotide polymorphisms. Biostatistics (2012), 13:2, 217-227.

Examples

```
m <- 10
h <- 0.2
n1 <- 50
pi_1 <- 0.5
alpha <- 0.01
rho=1
theta1 <- rep(0.3,m)
theta2 <- theta1 - h
PCC.n.optimal(n1, theta1, theta2, pi_1,alpha,rho)

################################################################3
data(Hapmap51)
n1 <- 50
pi_1 <- 0.5
theta1 <- Hapmap51$theta1
theta2 <- Hapmap51$theta2
alpha <- 0.1
```

```
rho=1
PCC.n.optimal(n1, theta1, theta2, pi_1,alpha,rho)
```

ROC.4Figures *The ROC figures for PCC(inf), PCC(n)_f, PCC(n)_MC, and PCC(n)_SVM.*

Description

The ROC figures for PCC(inf), PCC(n)_f, PCC(n)_MC, and PCC(n)_SVM.

Usage

```
ROC.4Figures(Plot,theta1,theta2,h,m,ll,n1,n2,low,high,bin_num,alpha,pi_1,pi_2,rho,kkk)
```

Arguments

Plot	If True, then the four figures will be plotted.
theta1	MAF for group 1.
theta2	MAF for group 2.
h	h=theta_1 - theta_2
m	Number of SNP's.
ll	Number of significan SNP's.
n1	Sample size in group 1.
n2	Sample size in group 2.
low	The lower bound for threshold K.
high	The upper bound for threshold K.
bin_num	Number of threshold K's, so the bin length is (high-low)/bin_num, and threshold is low + i*bin.
alpha	The significant level for SNP selection by Wald test.
pi_1	Percentage of group 1.
pi_2	Percentage of group 2.
rho	The percentage of significant SNP's.
kkk	The times of repeat in the simulation.

Details

The ROC figures for PCC(inf), PCC(n)_f, PCC(n)_MC, and PCC(n)_SVM.

Value

The sample size.

Author(s)

Xinyu Liu

References

Liu, X., et al. Sample size determination for classifiers based on single-nucleotide polymorphisms. Biostatistics (2012), 13:2, 217-227.

Examples

```
alpha = 0.1
rho = 1
kkk=200
low=-10
high=200
bin_num=5000
pi_1=pi_2=0.5

n1=n2=30
h=0.1
ll=m=10
theta1 <- rep(0.3,m)
theta2 <- theta1 - h
#ROC.4Figures(TRUE,theta1,theta2,h,m,ll,n1,n2,low,high,bin_num,alpha,pi_1,pi_2,rho,kkk)
#title("h=0.1, m=10, size=60")

n1=n2=200
h=0.1
ll=m=10
theta1 <- rep(0.3,m)
theta2 <- theta1 - h
#ROC.4Figures(TRUE,theta1,theta2,h,m,ll,n1,n2,low,high,bin_num,alpha,pi_1,pi_2,rho,kkk)
#title("h=0.1, m=10, size=200")

n1=n2=30
h=0.1
ll=m=50
theta1 <- rep(0.3,m)
theta2 <- theta1 - h
#ROC.4Figures(TRUE,theta1,theta2,h,m,ll,n1,n2,low,high,bin_num,alpha,pi_1,pi_2,rho,kkk)
#title("h=0.1, m=50, size=60")

n1=n2=200
h=0.1
ll=m=50
theta1 <- rep(0.3,m)
theta2 <- theta1 - h
#ROC.4Figures(TRUE,theta1,theta2,h,m,ll,n1,n2,low,high,bin_num,alpha,pi_1,pi_2,rho,kkk)
#title("h=0.1, m=50, size=200")

n1=n2=30
h=0.2
ll=m=10
theta1 <- rep(0.3,m)
theta2 <- theta1 - h
#ROC.4Figures(TRUE,theta1,theta2,h,m,ll,n1,n2,low,high,bin_num,alpha,pi_1,pi_2,rho,kkk)
#title("h=0.2, m=10, size=60")

n1=n2=30
h=0.2
```

```
ll=m=50
theta1 <- rep(0.3,m)
theta2 <- theta1 - h
#ROC.4Figures(TRUE,theta1,theta2,h,m,ll,n1,n2,low,high,bin_num,alpha,pi_1,pi_2,rho,kkk)
#title("h=0.2, m=50, size=60")
```

`ROC.infinity.plot` *The ROC plot for under the theoretical maximum PCC.*

Description

The ROC plot for under the theoretical maximum PCC.

Usage

```
ROC.infinity.plot(theta1, theta2, low=-200, high=200, bin_num=10000, lty=1, lwd=3, col="black", rh
```

Arguments

`theta1`	The MAF of case group.
`theta2`	The MAF of control group.
`low`	The low level of threshold K, default = -200.
`high`	The upper level of threshold K, default = 200.
`bin_num`	The number of bins while spanning the K, default = 10000.
`lty`	The style of line on the ROC, default=1.
`lwd`	The width of line on the ROC, default=3.
`col`	The color of the curve, default = "black".
`rho`	The percentage of SNPs with effect on case/control, default = 1.

Value

The ROC under PCC(infinity).

Author(s)

Xinyu Liu

References

Liu, X., et al. Sample size determination for classifiers based on single-nucleotide polymorphisms. Biostatistics (2012), 13:2, 217-227.

Examples

```
m <- 10
alpha = 0.01
rho = 1
theta1 <- rep(0.3,m)
h <- 0.2
theta2 <- theta1 - h
ROC.infinity.plot(theta1,theta2,alpha,rho=1)

##################################################################################
data(Hapmap51)
rho=1
ROC.infinity.plot(theta1 = Hapmap51$theta1,theta2=Hapmap51$theta2,low=-200, high=200, bin_num=10000, lty=1,
```

ROC.n.plot *The ROC plot under the linear classifier.*

Description

The ROC plot under the linear classifier.

Usage

```
ROC.n.plot(n1,n2,theta1,theta2,low=-200,high=200,bin_num=10000,alpha,lty=1,lwd=3,col="black",rhc
```

Arguments

n1	The number of case group.
n2	The number of control group.
theta1	The MAF of case group.
theta2	The MAF of control group.
low	The lower level of the K, default = -200.
high	The upper level of the K, default = 200.
bin_num	The number of bins in the span of K, default = 10000.
alpha	The significant level of choosing SNPs, default = 0.1.
lty	The style of the curve, default = 1.
lwd	The width of the curve, default =3.
col	The color of ROC, default = "black".
rho	The percentage of SNP with effect on case/control

Details

The ROC under the linear classifier.

Value

The ROC under the linear classifier.

Author(s)

Xinyu Liu

References

Liu, X., et al. Sample size determination for classifiers based on single-nucleotide polymorphisms. Biostatistics (2012), 13:2, 217-227.

Examples

```
m <- 10
h <- 0.2
alpha = 0.1
theta1 <- rep(0.3, m)
theta2 <- theta1 - h
n1 <- 50
n2 <- 50
ROC.n.plot(n1,n2,theta1,theta2,low=-200,high=200,bin_num=10000,alpha,lty=1,lwd=3,col="black",rho=1)

######################################################
data(Hapmap51)
n1 <- 50
n2 <- 50
alpha = 0.1
rho = 1
ROC.n.plot(n1,n2,theta1=Hapmap51$theta1,theta2=Hapmap51$theta2,low=-200,high=200,bin_num=10000,alpha,lty=1
```

`VUS.inf` *Compute the volumn under hypersurface (VUS) under theoretical maximum PCC, VUS(inf).*

Description

Compute the volumn under hypersurface (VUS) under theoretical maximum PCC, VUS(inf).

Usage

```
VUS.inf(C,ll,Theta,kk,abserr)
```

Arguments

`C`	Number of groups.
`ll`	Number of SNP's.
`Theta`	MAF matrix, with m rows and C columns.
`kk`	The number of random values generated to compute the integration by qhull method.
`abserr`	The cutoff of the error.

Details

Compute the volumn under hypersurface (VUS) under theoretical maximum PCC, VUS(inf).

Value

VUS(inf) value.

Author(s)

Xinyu Liu

References

Liu, X., et al. Sample size determination for classifiers based on single-nucleotide polymorphisms. Biostatistics (2012), 13:2, 217-227.

Examples

```
rho = 1
C=3
kk=100
U1=0.4
U2=0.49
Di=0.002
h = 0.1
ll = m = 50
abserr = 0.001
Theta <- Generate.Theta(C,m,U1,U2,h,Di,rho=1)$Theta
#VUS.inf(C,ll,Theta,kk,abserr) # 0.8685009
```

`VUS.n` *Compute the volumn under hypersurface (VUS) under linear classifier, VUS(n).*

Description

Compute the volumn under hypersurface (VUS) under linear classifier, VUS(n).

Usage

```
VUS.n(C,Theta,alpha,N,m,rho,kk,abserr)
```

Arguments

`C`	Number of groups.
`Theta`	MAF matrix with m rows and C columns.
`alpha`	The significant level for Wald test.
`N`	Matrix for sample size, e.g. matrix(c(n1,n2,n3,...,nC),nrow=C).
`m`	Number of SNP's.
`rho`	The percentage of SNP's with effect on different groups, default = 1.
`kk`	The number of random values generated to compute the integration by qhull method.
`abserr`	The cutoff of the error.

Details

Compute the volumn under hypersurface (VUS) under linear classifier, VUS(n).

Value

VUS(n) value.

Author(s)

Xinyu Liu

References

Liu, X., et al. Sample size determination for classifiers based on single-nucleotide polymorphisms. Biostatistics (2012), 13:2, 217-227.

Examples

```
rho = 1
alpha=0.01
C=3
kk=100
U1=0.4
U2=0.49
Di=0.002
h = 0.1
ll = m = 50
abserr = 0.001
n = 100
N <- matrix(n,nrow=C,ncol=1)
Theta <- Generate.Theta(C,m,U1,U2,h,Di,rho=1)$Theta
#VUS.inf(C,ll,Theta,kk,abserr)             #0.8686977
#VUS.n(C,Theta,alpha,N,m,rho,kk,abserr)     #0.4959834
```

Index

∗Topic **datasets**
Hapmap51, 11
Hapmap92, 12
HS348, 13

AUC.inf, 2
AUC.n, 3
AUC.Simulation, 4

Compute.MAF, 5

Find.SS.AUC, 6
Find.SS.PCC, 7
Find.SS.VUS, 9

Generate.Theta, 10

Hapmap51, 11
Hapmap92, 12
HS348, 13

PCC.inf.optimal, 14
PCC.n.optimal, 15

ROC.4Figures, 16
ROC.infinity.plot, 18
ROC.n.plot, 19

VUS.inf, 20
VUS.n, 21
