# Supplementary material for: Determination of sample size for a multi-class classifier based on single-nucleotide polymorphisms: a volume under the surface approach
Source: BMC Bioinformatics. 2014 Jun 14;15:190. doi: 10.1186/1471-2105-15-190 (PMC4071155; doi:10.1186/1471-2105-15-190)
Supplement: Additional file 3 — Appendix 1–5. [file 1471-2105-15-190-S3.pdf]

Appendix to Determination of sample size for a multi-class classifier based on single-nucleotide polymorphisms: A Volume Under the Surface approach

XINYU LIU
Department of Statistics
University of Georgia, Athens, GA 30602, USA.

YUPENG WANG
Computational Biology Service Unit
Cornell University, Ithaca, NY 14853

T.N. SRIRAM *
Department of Statistics
University of Georgia, Athens, GA 30602, USA.
tn@stat.uga.edu

December 19, 2013

*to whom correspondence should be addressed

In the following sections, the equation numbers without the prefix, A, correspond to those in the main article.

Appendix 1: Approximation for $PCC(\infty)$

To obtain an approximation for $PCC(\infty)$, fix k $(= 1, \ldots, D)$ and consider the $(D-1) \times 1$ vector $\vec{\mathbf{Y}}_{l,k}$ with components $Y_{l,k}(k') = \sum_{j=1}^{l} b^{j}_{k,k'} x_j$, for $k' \neq k$. Then, by the assumptions, the mean of $\vec{\mathbf{Y}}_{l,k}$, $\vec{\boldsymbol{\mu}}_{l,k} = E(\vec{\mathbf{Y}}_{l,k})$, is a $(D-1)\times 1$ vector with components $\mu_{l,k}(k') = \sum_{j=1}^{l} 2\theta_{k,j} b^{j}_{k,k'}$, and the Covariance of $\vec{\mathbf{Y}}_{l,k}$, $\boldsymbol{\Sigma}_{l,k} = Cov(\vec{\mathbf{Y}}_{l,k})$, is a $(D-1)\times(D-1)$ matrix with its (k', k'')-th element, $Cov(Y_{l,k}(k'), Y_{l,k}(k'')) = \sum_{j=1}^{l} 2\theta_{k,j}(1-\theta_{k,j}) b^{j}_{k,k'} b^{j}_{k,k''}$ for $k \neq k', k''$. Our aim is to show that, for large l

$$\vec{\mathbf{Y}}_{l,k} \approx \mathbf{N}(\vec{\boldsymbol{\mu}}_{l,k}, \boldsymbol{\Sigma}_{l,k}), \tag{A.1}$$

where $\mathbf{N}$ denotes the $(D-1)$-dimensional multivariate normal distribution. For each fixed $k = 1, 2, ..., D$, in order to prove the assertion, $\vec{\mathbf{Y}}_{l,k} \approx N(\vec{\boldsymbol{\mu}}_{l,k}, \boldsymbol{\Sigma}_{l,k})$ for large l, we will consider any linear combination $\vec{\boldsymbol{\beta}}\,'\vec{\mathbf{Y}}_{l,k}$ $(= \sum_{i=1, i\neq k}^{D} \beta_i Y_{l,k}(i))$ and first show that

$$\frac{\vec{\boldsymbol{\beta}}\,'\vec{\mathbf{Y}}_{l,k} - \vec{\boldsymbol{\beta}}\,'\vec{\boldsymbol{\mu}}_{l,k}}{\sqrt{\vec{\boldsymbol{\beta}}\,'\boldsymbol{\Sigma}_{l,k}\vec{\boldsymbol{\beta}}}} \Rightarrow N(0,1) \quad \text{as} \quad l \to \infty. \tag{A.2}$$

To this end, write

$$\vec{\boldsymbol{\beta}}\,'\vec{\mathbf{Y}}_{l,k} = \sum_{i=1, i\neq k}^{D} \beta_i Y_{l,k}(i) = \sum_{i=1, i\neq k}^{D} \sum_{j=1}^{l} \beta_i x_j b^{j}_{k,i} = \sum_{j=1}^{l} \Big(\sum_{i=1, i\neq k}^{D} \beta_i b^{j}_{k,i} \Big) x_j.$$

For a fixed $k = 1, \ldots, D$, set $Z_j = (\sum_{i=1, i\neq k}^{D} \beta_i b^{j}_{k,i}) x_j$ and note that $|Z_j| \leq M$ for some $M > 0$, because $|x_j| \leq 2$ and $|b^{j}_{k,i}| \leq log(99)$ since $\theta_{k,j}$ and $\theta_{i,j} \in (0.01, 0.5)$. Therefore, by the assumptions in Section 2.1, $\{Z_j\}$ is a sequence of independent and bounded random variables with $E(Z_j) = (\sum_{i=1, i\neq k}^{D} \beta_i b^{j}_{k,i}) 2\theta_{k,j}$ and $Var(Z_j) = (\sum_{i=1, i\neq k}^{D} \beta_i b^{j}_{k,i})^2 2\theta_{k,j}(1-\theta_{k,j})$, when $\vec{x} \in C_k$.

For any $(D-1)\times 1$ vector $\vec{\boldsymbol{\beta}} \neq \vec{\mathbf{0}}$, assume that $\sum_{j=1}^{l} \left[\sum_{k'=1, k'\neq k}^{D} \beta_{k'} \log\left(\frac{\theta_{k,j}(1-\theta_{k',j})}{\theta_{k',j}(1-\theta_{k,j})}\right) \right]^2 \to \infty$, as $l \to \infty$. Then, by this assumption and since $\theta_{k,j}(1-\theta_{k,j}) > 0.01 \times 0.5$, we have

$$\sum_{j=1}^{l} Var(Z_j) = 2 \sum_{j=1}^{l} \Big(\sum_{i=1, i\neq k}^{D} \beta_i b^{j}_{k,i} \Big)^2 \theta_{k,j}(1-\theta_{k,j}) > (0.01) \sum_{j=1}^{l} \Big(\sum_{i=1, i\neq k}^{D} \beta_i b^{j}_{k,i} \Big)^2 \to \infty,$$

as $l \to \infty$. Therefore, the desired result in (A.2) follows from Example 27-4 (also see Problem 27-4) of Billingsley (1995). Hence, by the Cramér-Wold device (see Billingsley, 1995, Theorem 29.4), we have that, for large l

$$\vec{\mathbf{Y}}_{l,k} \approx \mathbf{N}(\vec{\boldsymbol{\mu}}_{l,k}, \boldsymbol{\Sigma}_{l,k}),$$

This proves (A.1).

Note from equation (3) of the main article that $K_{k',k''} = -K_{k'',k'}$ and $\sum_{k=s}^{t} K_{k,(k+1)} = K_{s,t+1}$ for any $s, t = 1, \ldots, D-1$ with $s \leq t$. Let $K_{i,i+1} = K_i$ for $i = 1, ..., D-1$ and define

$$\vec{\mathbf{K}}_1 = \begin{pmatrix} K_1 \\ K_1 + K_2 \\ \cdots \\ K_1 + K_2 + \ldots + K_{D-1} \end{pmatrix}_{(D-1)\times 1} \tag{A.3}$$

Then, for $k = 2, ..., D$, it can be shown that

$$\vec{\mathbf{K}}_k = \vec{\mathbf{K}}_{(k-1)} - K_{(k-1)}\vec{\mathbf{1}} - K_{(k-1)}\vec{\mathbf{e}}_{(k-1)} \tag{A.4}$$

where $\vec{\mathbf{1}} = (1, 1, ..., 1)'$ and $\vec{\mathbf{e}}_{k-1} = (0, ..., 0, 1, 0, ..., 0)'$ with 1 in the $(k-1)$-th position and 0 elsewhere. Now, for any $(D-1) \times 1$ vector $\vec{\mathbf{K}}$, define

$$\tilde{\Phi}(\vec{\mathbf{K}}; \vec{\boldsymbol{\mu}}, \boldsymbol{\Sigma}) = \int_{\vec{\mathbf{K}}}^{\infty} \boldsymbol{\phi}(\vec{\mathbf{x}}; \vec{\boldsymbol{\mu}}, \boldsymbol{\Sigma}) d\vec{\mathbf{x}}, \tag{A.5}$$

where $\boldsymbol{\phi}$ is the $(D-1)$-dimensional multivariate normal density and $\int_{\vec{\mathbf{K}}}^{\infty}$ is a multiple integral. Then, we can conclude from (A.1) that for large l

$$\begin{aligned} PCC(\infty) &= \sum_{k=1}^{D} \pi_k P(\vec{\mathbf{Y}}_{l,k} > \vec{\mathbf{K}}_k | \vec{X} \in C_k) \\ &\approx \sum_{k=1}^{D} \pi_k \tilde{\Phi}(\vec{\mathbf{K}}_k; \vec{\boldsymbol{\mu}}_{l,k}, \boldsymbol{\Sigma}_{l,k}), \\ &= \sum_{k=1}^{D} \pi_k \int_{\vec{\mathbf{K}}_k}^{\infty} \boldsymbol{\phi}(\vec{\mathbf{x}}; \vec{\boldsymbol{\mu}}_{l,k}, \boldsymbol{\Sigma}_{l,k}) d\vec{\mathbf{x}} \end{aligned} \tag{A.6}$$

which establishes equation (4) of the main article.

Appendix 2: Wald test and its power function

Here, we derive a Wald test for testing $H_{0,j}^{k,k'} : \theta_{k,j} = \theta_{k',j}$ versus $H_{1,j}^{k,k'} : \theta_{k,j} \neq \theta_{k',j}$ for each j. For notational convenience, we let $k = 1$ and $k' = 2$, and $\theta_{1,j} = \theta_1$ and $\theta_{2,j} = \theta_2$ for the derivations below. For each X_j satisfying Assumption 2 stated in the Methods section of the main article, let $n_{1k} = \sum_{j=1}^{n_k} I_{\{x_j=0\}}$, $n_{2k} = \sum_{j=1}^{n_k} I_{\{x_j=1\}}$ and $n_{3k} = \sum_{j=1}^{n_k} I_{\{x_j=2\}}$ with $\sum_{i=1}^{3} n_{ik} = n_k$ for $k = 1, 2$. Then, by Assumption 2 and the independence of the two classes, the likelihood function for a sample of size n_k from each class is:

$$L(\theta_1, \theta_2) = \prod_{k=1}^{2} [(1-\theta_k)^2]^{n_{1k}} [2\theta_k(1-\theta_k)]^{n_{2k}} [\theta_k^2]^{n_{3k}}.$$

Maximizing the log-likelihood, $\log L(\theta_1, \theta_2)$, with respect to (θ_1, θ_2), it can be shown that the maximum likelihood estimator (MLE) of θ_1 and θ_2 are, respectively:

$$\hat{\theta}_1 = \frac{n_{21} + 2n_{31}}{2n_1} \quad \text{and} \quad \hat{\theta}_2 = \frac{n_{22} + 2n_{32}}{2n_2}. \tag{A.7}$$

Also, the Fisher information matrix at (θ_1, θ_2) for $n_k = 1$ is $I(\theta_1, \theta_2) = \begin{pmatrix} \frac{2}{\theta_1(1-\theta_1)} & 0 \\ 0 & \frac{2}{\theta_2(1-\theta_2)} \end{pmatrix}$. Let $2n = n_1 + n_2$. Then, by the asymptotic normality of the MLE, it follows that $\sqrt{n}(\hat{\theta}_1 - \theta_1, \hat{\theta}_2 - \theta_2)' \xrightarrow{d} N_2(\mathbf{0}, I^{-1}(\theta_1, \theta_2))$. Now, since $g(\theta_1, \theta_2) = \theta_1 - \theta_2$ is differentiable at (θ_1, θ_2), it follows from the delta method that $\sqrt{n}[g(\hat{\theta}_1, \hat{\theta}_2) - g(\theta_1, \theta_2)] \xrightarrow{d} N(0, \frac{\theta_1(1-\theta_1)+\theta_2(1-\theta_2)}{2})$. Therefore, under $H_0 : \theta_1 = \theta_2$, the Wald test statistic

$$Q_2 = \frac{2n(\hat{\theta}_1 - \hat{\theta}_2)^2}{\hat{\theta}_1(1-\hat{\theta}_1) + \hat{\theta}_2(1-\hat{\theta}_2)} \xrightarrow{d} \chi_1^2 \quad \text{as} \quad n \to \infty, \tag{A.8}$$

where χ_1^2 has chi-square distribution with 1 degree of freedom. However, under $H_a : \theta_1 \neq \theta_2$, say $\theta_1 - \theta_2 = h$, it follows from the above arguments that $Q_2 \xrightarrow{d} \chi_1^2(\lambda^2)$, where $\chi_1^2(\lambda^2)$ has non-central chi-square distribution with the non-centrality parameter, $\lambda^2 = 2nh^2/[\theta_1(1-\theta_1) + (\theta_1 - h)(1 - \theta_1 + h)]$. Therefore, the power of the Wald test (when $\theta_1 - \theta_2 = h \neq 0$) is:

$$1 - \beta(n_1, n_2, h) \approx P\left(\chi_1^2(\lambda^2) > \chi_{1,(1-\alpha)}^2\right),$$

where $\chi_{1,(1-\alpha)}^2$ is the $(1-\alpha)$ percentile of χ_1^2. For ease of presentation, we had suppressed the subscript j. For each $j = 1, \ldots, m$, the power of the Wald test for $H_0 : \theta_{k,j} = \theta_{k',j}$ versus $H_1 : \theta_{k,j} \neq \theta_{k',j}$ at $\theta_{k,j} = \theta_{k',j} + h_j$ is denoted by $1 - \beta_j^{k,k'}(n_k, n_{k'}, h_j)$. Note that the power,

$1-\beta_j^{k,k'}(n_k, n_{k'}, h_j)$, of the test is determined using a non-central Chi-square distribution with a non-centrality parameter, which depends on $n_k + n_{k'}$ and h_j.

Appendix 3: Approximation for $PCC(\vec{n})$

To obtain an approximation for $PCC(\vec{n})$, we adopt the same approach as in Appendix 1. For the linear classifier given in (5) of the main article, consider the $(D-1)\times 1$ vector $\tilde{\vec{\mathbf{Y}}}_{n,m,k}$ with components $\tilde{Y}_{n,m,k}(k') = \sum_{j=1}^{m} \hat{b}^j_{k,k'} w_{j,n}(k,k')x_j$, for $k' \neq k$. Then, using the assumptions made in the Methods section of the main article and that $(\hat{b}^j_{k,k'} - b^j_{k,k'}) = O(n^{-1/2})$, it is shown below that the mean, $E(\tilde{\vec{\mathbf{Y}}}_{n,m,k}) = \tilde{\vec{\boldsymbol{\mu}}}_{m,k}$, is a $(D-1)\times 1$ vector with components $\tilde{\vec{\mu}}_{m,k}(k') \approx \sum_{j=1}^{m} 2\theta_{k,j} b^j_{k,k'} \tilde{\eta}_j^{k,k'}$. In addition, we also compute below an approximate expression for the $(D-1)\times(D-1)$ Covariance matrix of $\tilde{\vec{\mathbf{Y}}}_{n,m,k}$, denoted by $\tilde{\mathbf{\Sigma}}_{m,k}$.

First, note from the calculations carried out in Appendix 2 that

$$\begin{aligned} P(Reject\ H_{0,j}^{k,k'}) &= P(Reject\ H_{0,j}^{k,k'}|H_{0,j}^{k,k'})P(H_{0,j}^{k,k'}) \\ &\quad + P(Reject\ H_{0,j}^{k,k'}|H_{1,j}^{k,k'})P(H_{1,j}^{k,k'}) \\ &= [\rho\{1-\beta_j^{k,k'}(n_k, n_{k'}, h)\} + (1-\rho)\alpha] = \tilde{\eta}_j^{k,k'}, \end{aligned} \tag{A.9}$$

where ρ is from Assumption 3 of the main article and $\tilde{\eta}_j^{k,k'}$ depends on $(n_k, n_{k'})$. Therefore, from the definition of $w_{j,n}(k,k')$ in the linear classifier,

$$E(w_{j,n}(k,k')) = E((w_{j,n}(k,k'))^2) = P(Reject\ H_{0,j}^{k,k'}) = \tilde{\eta}_j^{k,k'}.$$

From these, it can be shown that

$$\begin{aligned} E(\hat{b}^j_{k,k'} w_{j,n}(k,k')x_j) &= E\{E(\hat{b}^j_{k,k'} w_{j,n}(k,k')x_j)|w_{j,n}(k,k')\} \\ &\approx E(2\theta_{k,j}\hat{b}^j_{k,k'} w_{j,n}(k,k')) \\ &\approx 2\theta_{k,j} b^j_{k,k'} \tilde{\eta}_j^{k,k'} \end{aligned}$$

and

$$
\begin{aligned}
&Cov(w_{j,n}(k,k')\hat{b}^j_{k,k'}x_j, w_{j,n}(k,k'')\hat{b}^j_{k,k'}x_j)\\
&= E(x_j^2 w_{j,n}(k,k')w_{j,n}(k,k'')\hat{b}^j_{k,k'}\hat{b}^j_{k,k''}) - E(w_{j,n}(k,k')\hat{b}^j_{k,k'}x_j)E(w_{j,n}(k,k'')\hat{b}^j_{k,k''}x_j)\\
&= E\{E[x_j^2\hat{b}^j_{k,k'}\hat{b}^j_{k,k''}w_{j,n}(k,k')w_{j,n}(k,k'')|w_{j,n}(k,k')w_{j,n}(k,k'')]\} - E(w_{j,n}(k,k')\hat{b}^j_{k,k'}x_j)E(w_{j,n}(k,k'')\hat{b}^j_{k,k''}x_j)\\
&= E\{w_{j,n}(k,k')\hat{b}^j_{k,k'}\hat{b}^j_{k,k'}w_{j,n}(k,k'')[2\theta_{k,j}(1-\theta_{k,j})+4\theta_{k,j}^2]\} - E(w_{j,n}(k,k')\hat{b}^j_{k,k'}x_j)E(w_{j,n}(k,k'')\hat{b}^j_{k,k''}x_j)\\
&\approx \begin{cases} (b^j_{k,k'})^2[2\theta_{k,j}(1-\theta_{k,j})\tilde{\eta}_j^{k,k'} + 4\theta_{k,j}^2\tilde{\eta}_j^{k,k'}(1-\tilde{\eta}_j^{k,k'})], \text{if } k'=k'' \\ [2\theta_{k,j}(1-\theta_{k,j})+4\theta_{k,j}^2]b^j_{k,k'}b^j_{k,k''}E(w_{j,n}(k,k')w_{j,n}(k,k'')) - (2\theta_{k,j}b^j_{k,k'}\tilde{\eta}_j^{k,k'})(2\theta_{k,j}b^j_{k,k''}\tilde{\eta}_j^{k,k''}),\ \text{if } k'\neq k''. \end{cases}
\end{aligned}
\tag{A.10}
$$

Now, we give an approximation for $E(w_{j,n}(k,k')w_{j,n}(k,k''))$ in (A.10). First, note using the results in Appendix 2 that $\sqrt{2n}(\hat{\theta}_{i,j}-\theta_{i,j}) \Rightarrow N(0,\theta_{i,j}(1-\theta_{i,j}))$ where $i=k,k',k''$ and $\sqrt{2n}(\hat{\theta}_{k,j}-\hat{\theta}_{s,j}-\theta_{k,j}+\theta_{s,j}) \Rightarrow N(0,\theta_{k,j}(1-\theta_{k,j})+\theta_{s,j}(1-\theta_{s,j}))$, where $s=k',k''$. Now define $T_1 \triangleq \frac{\sqrt{2n}(\hat{\theta}_{k,j}-\hat{\theta}_{k',j})}{\sqrt{\theta_{k,j}(1-\theta_{k,j})+\theta_{k',j}(1-\theta_{k',j})}}$ and $T_2 \triangleq \frac{\sqrt{2n}(\hat{\theta}_{k,j}-\hat{\theta}_{k'',j})}{\sqrt{\theta_{k,j}(1-\theta_{k,j})+\theta_{k'',j}(1-\theta_{k'',j})}}$. Then from (A.8), we have $Q_2(\theta_{k,j},\theta_{k',j}) \triangleq T_1^2$ and $Q_2(\theta_{k,j},\theta_{k'',j}) \triangleq T_2^2$ and recall that $H_{0,j}^{k,k'}: \theta_{k,j}=\theta_{k',j}$ and $H_{0,j}^{k,k''}: \theta_{k,j}=\theta_{k'',j}$. From these, we have

$$
\begin{aligned}
&E(w_{j,n}(k,k')w_{j,n}(k,k''))\\
&= P(reject\ H_{0,j}^{k,k'} \cap reject\ H_{0,j}^{k,k''})\\
&= P(\{Q_2(\theta_{k,j},\theta_{k',j}) > \chi^2_{1-\alpha}(1)\} \cap \{Q_2(\theta_{k,j},\theta_{k'',j}) > \chi^2_{1-\alpha}(1)\})\\
&= P(|T_1| > \sqrt{\chi^2_{1-\alpha}(1)} \cap |T_2| > \sqrt{\chi^2_{1-\alpha}(1)}).
\end{aligned}
$$

For k,k',k'', we can mimic the arguments leading to (A.7) and (A.8), and show that (T_1,T_2) is asymptotically multivariate normal. Note that the means, variances, and covariances of T_1 and T_2 are given by:

$$E(T_1) = \frac{\sqrt{2n}(\theta_{k,j}-\theta_{k',j})}{\sqrt{\theta_{k,j}(1-\theta_{k,j})+\theta_{k',j}(1-\theta_{k',j})}}$$

$$E(T_2) = \frac{\sqrt{2n}(\theta_{k,j}-\theta_{k'',j})}{\sqrt{\theta_{k,j}(1-\theta_{k,j})+\theta_{k'',j}(1-\theta_{k'',j})}}$$

$$Var(T_1) = Var(T_2) = 1$$

$$Cov(T_1, T_2) = \frac{\theta_{k,j}(1-\theta_{k,j})}{\sqrt{[\theta_{k,j}(1-\theta_{k,j}) + \theta_{k',j}(1-\theta_{k',j})][\theta_{k,j}(1-\theta_{k,j}) + \theta_{k'',j}(1-\theta_{k'',j})]}}.$$

Returning to the approximation of $PCC(\vec{n})$, assume for simplicity that $\tilde{K}_{k,k'}$ in (5) of the main article also satisfies the same properties as $K_{k,k'}$ in (A.3) and (A.4); that is, $\tilde{K}_{k',k''} = -\tilde{K}_{k'',k'}$ and $\sum_{k=s}^{t} \tilde{K}_{k,(k+1)} = \tilde{K}_{s,t+1}$ for any $s, t = 1, \ldots, D-1$ with $s \leq t$. Let $\tilde{K}_{i,i+1} = \tilde{K}_i$ for $i = 1, ..., D-1$, and define $\tilde{\vec{\mathbf{K}}}_1$ and $\tilde{\vec{\mathbf{K}}}_k$ as in (A.3) and (A.4), respectively. Then, once again, as in (A.1), for large m we can show that $\tilde{\vec{\mathbf{Y}}}_{n,m,k} \approx \mathbf{N}(\tilde{\vec{\boldsymbol{\mu}}}_{m,k}, \tilde{\boldsymbol{\Sigma}}_{m,k})$.

Then, as in (A.5) and (A.6), the PCC of the linear classifier is:

$$\begin{aligned} PCC(\vec{n}) &= \sum_{k=1}^{D} \pi_k P(\tilde{\vec{\mathbf{Y}}}_{n,m,k} > \tilde{\vec{\mathbf{K}}}_k | \vec{X} \in C_k) \\ &\approx \sum_{k=1}^{D} \pi_k \tilde{\Phi}(\tilde{\vec{\mathbf{K}}}_k; \tilde{\vec{\boldsymbol{\mu}}}_{m,k}, \tilde{\boldsymbol{\Sigma}}_{m,k}) \\ &= \sum_{k=1}^{D} \pi_k \int_{\tilde{\vec{\mathbf{K}}}_k}^{\infty} \boldsymbol{\phi}(\vec{\mathbf{x}}; \tilde{\vec{\boldsymbol{\mu}}}_{l,k}, \tilde{\boldsymbol{\Sigma}}_{l,k}) d\vec{\mathbf{x}} \qquad \text{(A.11)} \end{aligned}$$

Note that $PCC(\vec{n})$ depends on $\vec{n} = (n_1, \ldots, n_D)'$ through $(\tilde{\vec{\boldsymbol{\mu}}}_{m,k}, \tilde{\boldsymbol{\Sigma}}_{m,k})$, which depend on $\{\tilde{\eta}_j^{k,k'}; k, k' = 1, \ldots, D\}$.

Appendix 4: PCC and VUS expressions for three classes

Here, we assume that $D = 3$ and obtain an expression for $PCC(\infty), PCC(\vec{n})$ and $VUS(\infty)$.

Calculation of $PCC(\infty)$:

$$\begin{aligned} PCC(\infty) = {} & \pi_1 P(\sum_{j=1}^{l} x_j b_{1,2}^j > K_{1,2}, \ \sum_{j=1}^{l} x_j b_{1,3}^j > K_{1,3}) + \pi_2 P(\sum_{j=1}^{l} x_j b_{2,1}^j > K_{2,1}, \ \sum_{j=1}^{l} x_j b_{2,3}^j > K_{2,3}) \\ & + \pi_3 P(\sum_{j=1}^{l} x_j b_{3,1}^j > K_{3,1}, \ \sum_{j=1}^{l} x_j b_{3,2}^j > K_{3,2}). \end{aligned}$$

Let

$$
\begin{aligned}
K_1 &\triangleq K_{1,2} = -K_{2,1} \\
K_2 &\triangleq K_{2,3} = -K_{3,2} \\
K_{1,3} &= K_1 + K_2 \\
K_{3,1} &= -(K_1 + K_2).
\end{aligned}
$$

Then, rewrite $PCC(\infty)$ as

$$
\begin{aligned}
PCC(\infty) = \pi_1 P(\sum_{j=1}^{l} x_j b_{1,2}^j > K_1,\ \sum_{j=1}^{l} x_j b_{1,3}^j > (K_1 + K_2)) + \pi_2 P(\sum_{j=1}^{l} x_j b_{2,1}^j > -K_1,\ \sum_{j=1}^{l} x_j b_{2,3}^j > K_2) \\
+ \pi_3 P(\sum_{j=1}^{l} x_j b_{3,1}^j > -(K_1 + K_2),\ \sum_{j=1}^{l} x_j b_{3,2}^j > -K_2) \\
\approx \pi_1 \tilde{\Phi}((K_1, K_1 + K_2)'; \vec{\boldsymbol{\mu}}_{l,1}, \boldsymbol{\Sigma}_{l,1}) + \pi_2 \tilde{\Phi}((-K_1, K_2)'; \vec{\boldsymbol{\mu}}_{l,2}, \boldsymbol{\Sigma}_{l,2}) \\
+ \pi_3 \tilde{\Phi}((-(K_1 + K_2), -K_2)'; \vec{\boldsymbol{\mu}}_{l,3}, \boldsymbol{\Sigma}_{l,3}), \qquad \text{(A.12)}
\end{aligned}
$$

where

$$
\vec{\boldsymbol{\mu}}_{l,1} = \begin{pmatrix} \sum_{j=1}^{l} 2\theta_{1,j} b_{1,2}^j \\ \sum_{j=1}^{l} 2\theta_{1,j} b_{1,3}^j \end{pmatrix}
$$

$$
\vec{\boldsymbol{\mu}}_{l,2} = \begin{pmatrix} \sum_{j=1}^{l} 2\theta_{2,j} b_{2,1}^j \\ \sum_{j=1}^{l} 2\theta_{2,j} b_{2,3}^j \end{pmatrix}
$$

$$
\vec{\boldsymbol{\mu}}_{l,3} = \begin{pmatrix} \sum_{j=1}^{l} 2\theta_{3,j} b_{3,1}^j \\ \sum_{j=1}^{l} 2\theta_{3,j} b_{3,2}^j \end{pmatrix}
$$

$$
\boldsymbol{\Sigma}_{l,1} \triangleq \begin{pmatrix} \sum_{j=1}^{l} 2(b_{1,2}^j)^2 \theta_{1,j}(1-\theta_{1,j}) & \sum_{j=1}^{l} 2b_{1,2}^j b_{1,3}^j \theta_{1,j}(1-\theta_{1,j}) \\ \sum_{j=1}^{l} 2b_{1,3}^j b_{1,2}^j \theta_{1,j}(1-\theta_{1,j}) & \sum_{j=1}^{l} 2(b_{1,3}^j)^2 \theta_{1,j}(1-\theta_{1,j}) \end{pmatrix}
$$

$$
\boldsymbol{\Sigma}_{l,2} \triangleq \begin{pmatrix} \sum_{j=1}^{l} 2(b_{2,1}^j)^2 \theta_{2,j}(1-\theta_{2,j}) & \sum_{j=1}^{l} 2b_{2,1}^j b_{2,3}^j \theta_{2,j}(1-\theta_{2,j}) \\ \sum_{j=1}^{l} 2b_{2,3}^j b_{2,1}^j \theta_{2,j}(1-\theta_{2,j}) & \sum_{j=1}^{l} 2(b_{2,3}^j)^2 \theta_{2,j}(1-\theta_{2,j}) \end{pmatrix}
$$

$$\mathbf{\Sigma}_{l,3} \triangleq \begin{pmatrix} \sum_{j=1}^{l} 2(b_{3,1}^{j})^2\theta_{3,j}(1-\theta_{3,j}) & \sum_{j=1}^{l} 2b_{3,1}^{j}b_{3,2}^{j}\theta_{3,j}(1-\theta_{3,j}) \\ \sum_{j=1}^{l} 2b_{3,1}^{j}b_{3,2}^{j}\theta_{3,j}(1-\theta_{3,j}) & \sum_{j=1}^{l} 2(b_{3,2}^{j})^2\theta_{3,j}(1-\theta_{3,j}) \end{pmatrix}.$$

Calculation of $PCC(\vec{n})$:

Note from (5) of the main article that

$$\vec{X} \in C_1 \ if \ \{\sum_{j=1}^{m} \hat{b}_{1,2}^{j} w_{j,n}(1,2)x_j > \tilde{K_1}\} \ \& \ \{\sum_{j=1}^{m} \hat{b}_{1,3}^{j} w_{j,n}(1,3)x_j > \tilde{K_1} + \tilde{K_2}\},$$

$$\vec{X} \in C_2 \ if \ \{\sum_{j=1}^{m} \hat{b}_{2,1}^{j} w_{j,n}(2,1)x_j > -\tilde{K_1}\} \ \& \ \{\sum_{j=1}^{m} \hat{b}_{2,3}^{j} w_{j,n}(2,3)x_j > \tilde{K_2}\},$$

$$\vec{X} \in C_3 \ if \ \{\sum_{j=1}^{m} \hat{b}_{3,1}^{j} w_{j,n}(3,1)x_j > -(\tilde{K_1} + \tilde{K_2})\} \ \& \ \{\sum_{j=1}^{m} \hat{b}_{3,2}^{j} w_{j,n}(3,2)x_j > -\tilde{K_2}\}.$$

Therefore,

$$\begin{aligned} PCC(\vec{n}) &= \pi_1 P(\sum_{j=1}^{m} \hat{b}_{1,2}^{j} w_{j,n}(1,2)x_j > \tilde{K_1}, \ \sum_{j=1}^{m} \hat{b}_{1,3}^{j} w_{j,n}(1,3)x_j > \tilde{K_1} + \tilde{K_2}) \\ &\quad + \pi_2 P(\sum_{j=1}^{m} \hat{b}_{2,1}^{j} w_{j,n}(2,1)x_j > -\tilde{K_1}, \ \sum_{j=1}^{m} \hat{b}_{2,3}^{j} w_{j,n}(2,3)x_j > \tilde{K_2}) \\ &\quad + \pi_3 P(\sum_{j=1}^{m} \hat{b}_{3,1}^{j} w_{j,n}(3,1)x_j > -(\tilde{K_1} + \tilde{K_2}), \ \sum_{j=1}^{m} \hat{b}_{3,2}^{j} w_{j,n}(3,2)x_j > -\tilde{K_2}) \\ &\approx \pi_1 \tilde{\Phi}((\tilde{K_1}, \tilde{K_1} + \tilde{K_2})'; \tilde{\vec{\boldsymbol{\mu}}}_{l,1}, \tilde{\mathbf{\Sigma}}_{l,1}) + \pi_2 \tilde{\Phi}((-\tilde{K_1}, \tilde{K_2})'; \tilde{\vec{\boldsymbol{\mu}}}_{l,2}, \tilde{\mathbf{\Sigma}}_{l,2}) \\ &\quad + \pi_3 \tilde{\Phi}((-(\tilde{K_1} + \tilde{K_2}), -\tilde{K_2})'; \tilde{\vec{\boldsymbol{\mu}}}_{l,3}, \tilde{\mathbf{\Sigma}}_{l,3}), \end{aligned}$$

where

$$\tilde{\vec{\boldsymbol{\mu}}}_{l,1} = \begin{pmatrix} 2\sum_{j=1}^{m} \theta_{1,j}\tilde{\eta}_j^{1,2} \\ 2\sum_{j=1}^{m} \theta_{1,j}\tilde{\eta}_j^{1,3} \end{pmatrix}$$

$$\tilde{\vec{\mu}}_{l,2} = \begin{pmatrix} 2\sum_{j=1}^{m}\theta_{2,j}\tilde{\eta}_j^{2,1} \\ 2\sum_{j=1}^{m}\theta_{2,j}\tilde{\eta}_j^{2,3} \end{pmatrix}$$

$$\tilde{\vec{\mu}}_{l,3} = \begin{pmatrix} 2\sum_{j=1}^{m}\theta_{3,j}\tilde{\eta}_j^{3,1} \\ 2\sum_{j=1}^{m}\theta_{3,j}\tilde{\eta}_j^{3,2} \end{pmatrix},$$

and the 2×2 variance-covariance matrices (written in a vector form due to the length of each expression) are given by:

$$\tilde{\mathbf{\Sigma}}_{l,1} = \begin{pmatrix} \sum_{j=1}^{m}(b_{1,2}^{j})^2[2\theta_{1,j}(1-\theta_{1,j})\tilde{\eta}_j^{1,2} + 4\theta_{1,2}^2\tilde{\eta}_j^{1,2}(1-\tilde{\eta}_j^{1,2})] \\ \sum_{j=1}^{m}[2\theta_{1,j}(1-\theta_{1,j}) + 4\theta_{1,j}^2]b_{1,2}^{j}b_{1,3}^{j}E(w_{j,n}(1,2)w_{j,n}(1,3)) - \sum_{j=1}^{m}4\theta_{1,j}^2 b_{1,2}^{j}b_{1,3}^{j}\tilde{\eta}_j^{1,2}\tilde{\eta}_j^{1,3}] \\ \sum_{j=1}^{m}[2\theta_{1,j}(1-\theta_{1,j}) + 4\theta_{1,j}^2]b_{1,2}^{j}b_{1,3}^{j}E(w_{j,n}(1,2)w_{j,n}(1,3)) - \sum_{j=1}^{m}4\theta_{1,j}^2 b_{1,2}^{j}b_{1,3}^{j}\tilde{\eta}_j^{1,2}\tilde{\eta}_j^{1,3}] \\ \sum_{j=1}^{m}(b_{1,3}^{j})^2[2\theta_{1,j}(1-\theta_{1,j})\tilde{\eta}_j^{1,3} + 4\theta_{1,3}^2\tilde{\eta}_j^{1,3}(1-\tilde{\eta}_j^{1,3})] \end{pmatrix}$$

$$\tilde{\mathbf{\Sigma}}_{l,2} = \begin{pmatrix} \sum_{j=1}^{m}(b_{2,1}^{j})^2[2\theta_{2,j}(1-\theta_{2,j})\tilde{\eta}_j^{2,1} + 4\theta_{2,1}^2\tilde{\eta}_j^{2,1}(1-\tilde{\eta}_j^{2,1})] \\ \sum_{j=1}^{m}[2\theta_{2,j}(1-\theta_{2,j}) + 4\theta_{2,j}^2]b_{2,1}^{j}b_{2,3}^{j}E(w_{j,n}(2,1)w_{j,n}(2,3)) - \sum_{j=1}^{m}4\theta_{2,j}^2 b_{2,1}^{j}b_{2,3}^{j}\tilde{\eta}_j^{2,1}\tilde{\eta}_j^{2,3}] \\ \sum_{j=1}^{m}[2\theta_{2,j}(1-\theta_{2,j}) + 4\theta_{2,j}^2]b_{2,2}^{j}b_{2,3}^{j}E(w_{j,n}(2,1)w_{j,n}(2,3)) - \sum_{j=1}^{m}4\theta_{2,j}^2 b_{2,1}^{j}b_{2,3}^{j}\tilde{\eta}_j^{2,1}\tilde{\eta}_j^{2,3}] \\ \sum_{j=1}^{m}(b_{2,3}^{j})^2[2\theta_{2,j}(1-\theta_{2,j})\tilde{\eta}_j^{2,3} + 4\theta_{2,3}^2\tilde{\eta}_j^{2,3}(1-\tilde{\eta}_j^{2,3})] \end{pmatrix}$$

$$\tilde{\mathbf{\Sigma}}_{l,3} = \begin{pmatrix} \sum_{j=1}^{m}(b_{3,1}^{j})^2[2\theta_{3,j}(1-\theta_{3,j})\tilde{\eta}_j^{3,2} + 4\theta_{3,j}^2\tilde{\eta}_j^{3,2}(1-\tilde{\eta}_j^{3,2})] \\ \sum_{j=1}^{m}[2\theta_{3,j}(1-\theta_{3,j}) + 4\theta_{3,j}^2]b_{3,1}^{j}b_{3,2}^{j}E(w_{j,n}(3,1)w_{j,n}(3,2)) - \sum_{j=1}^{m}4\theta_{3,j}^2 b_{3,1}^{j}b_{3,2}^{j}\tilde{\eta}_j^{3,1}\tilde{\eta}_j^{3,2}] \\ \sum_{j=1}^{m}[2\theta_{3,j}(1-\theta_{3,j}) + 4\theta_{3,j}^2]b_{3,1}^{j}b_{3,2}^{j}E(w_{j,n}(3,1)w_{j,n}(3,2)) - \sum_{j=1}^{m}4\theta_{3,j}^2 b_{3,1}^{j}b_{3,2}^{j}\tilde{\eta}_j^{3,1}\tilde{\eta}_j^{3,2}] \\ \sum_{j=1}^{m}(b_{3,2}^{j})^2[2\theta_{3,j}(1-\theta_{3,j})\tilde{\eta}_j^{3,1} + 4\theta_{3,j}^2\tilde{\eta}_j^{3,2}(1-\tilde{\eta}_j^{3,2})] \end{pmatrix}.$$

Calculation of $VUS(\infty)$ and $VUS(\vec{n})$:

If we denote $N_2(x_1, x_2; \vec{\boldsymbol{\mu}}, \mathbf{\Sigma})$ as the two-dimensional normal density function with mean $\vec{\boldsymbol{\mu}}$ and variance-covariance matrix $\mathbf{\Sigma}$, then from (A.12) and (7) of the main article, the right side of $PCC(\infty)$ involves

$$\xi_{1,1} = \int_{K_1}^{\infty} \int_{K_1+K_2}^{\infty} N_2(x_1, x_2; \vec{\boldsymbol{\mu}}_{l,1}, \boldsymbol{\Sigma}_{l,1}) dx_1 dx_2$$
$$\xi_{2,2} = \int_{-K_1}^{\infty} \int_{K_2}^{\infty} N_2(x_1, x_2; \vec{\boldsymbol{\mu}}_{l,2}, \boldsymbol{\Sigma}_{l,2}) dx_1 dx_2$$
$$\xi_{3,3} = \int_{-K_1-K_2}^{\infty} \int_{-K_2}^{\infty} N_2(x_1, x_2; \vec{\boldsymbol{\mu}}_{l,3}, \boldsymbol{\Sigma}_{l,3}) dx_1 dx_2.$$

Then, from (9) of the main article we have

$$VUS(\infty) = \int_0^1 \int_0^1 \xi_{1,1}(K_1, K_2) d\xi_{2,2}(K_1, K_2) d\xi_{3,3}(K_1, K_2).$$

Similarly, we can derive expressions for $VUS(\vec{n})$.

Appendix 5: Monte Carlo Simulations using AUC

To compare the performance of our linear classifier with another classifier in the literature, such as the SVM, we also computed the $AUC(n)$ values corresponding to the SVM for the same simulation setup as the one described in Table 1 of Liu et al. (2012). For *ROC* and *AUC* calculations, we consider the special case, $\vec{\theta_1} = (\theta_1, \ldots, \theta_1)'$ and $\vec{\theta_2} = (\theta_2, \ldots, \theta_2)'$ with $\theta_1 > \theta_2$. These are given in Figure 1 below. Note that, unlike our linear classifier, there is no approximate formula available to calculate the $AUC(n)$ for SVM. Therefore, we cannot compare $AUC(n)$ values for our linear classifier (or the $AUC(\infty)$ values) with $AUC(n)$ values for SVM. Figure 1 shows that the *ROC*_MC values are essentially same as those for the *ROC* for the SVM_MC. This says that our linear classifier is as good as or slightly better than the SVM. Table 1 compares the approximate values of $AUC(n)$, denoted by $AU\hat{C}(n)$, with the Monte Carlo based estimates, $AU\hat{C}(n)$MC, for various specifications. To obtain $AU\hat{C}(n)$MC values, for each specification in Table 1, we simulated a *training* data and a *testing* data of SNPs, each having the same sample sizes. The training data was used to build the linear classifier, while the testing data was used to determine the frequency of correct classification of the linear classifier. This process was repeated 200 times in order to compute the average correct classification frequency, $AU\hat{C}(n)$MC, given in Table 1. It is evident from Table 1 that the $B\hat{i}as = AU\hat{C}(n)\text{MC} - AU\hat{C}(n)$ is negligible in most cases, thereby validating the use of our approximation for $AUC(n)$. Also note that both $AU\hat{C}(n)$MC and $AU\hat{C}(n)$ are close to $AU\hat{C}(\infty)$, approximate values of $AUC(\infty)$.

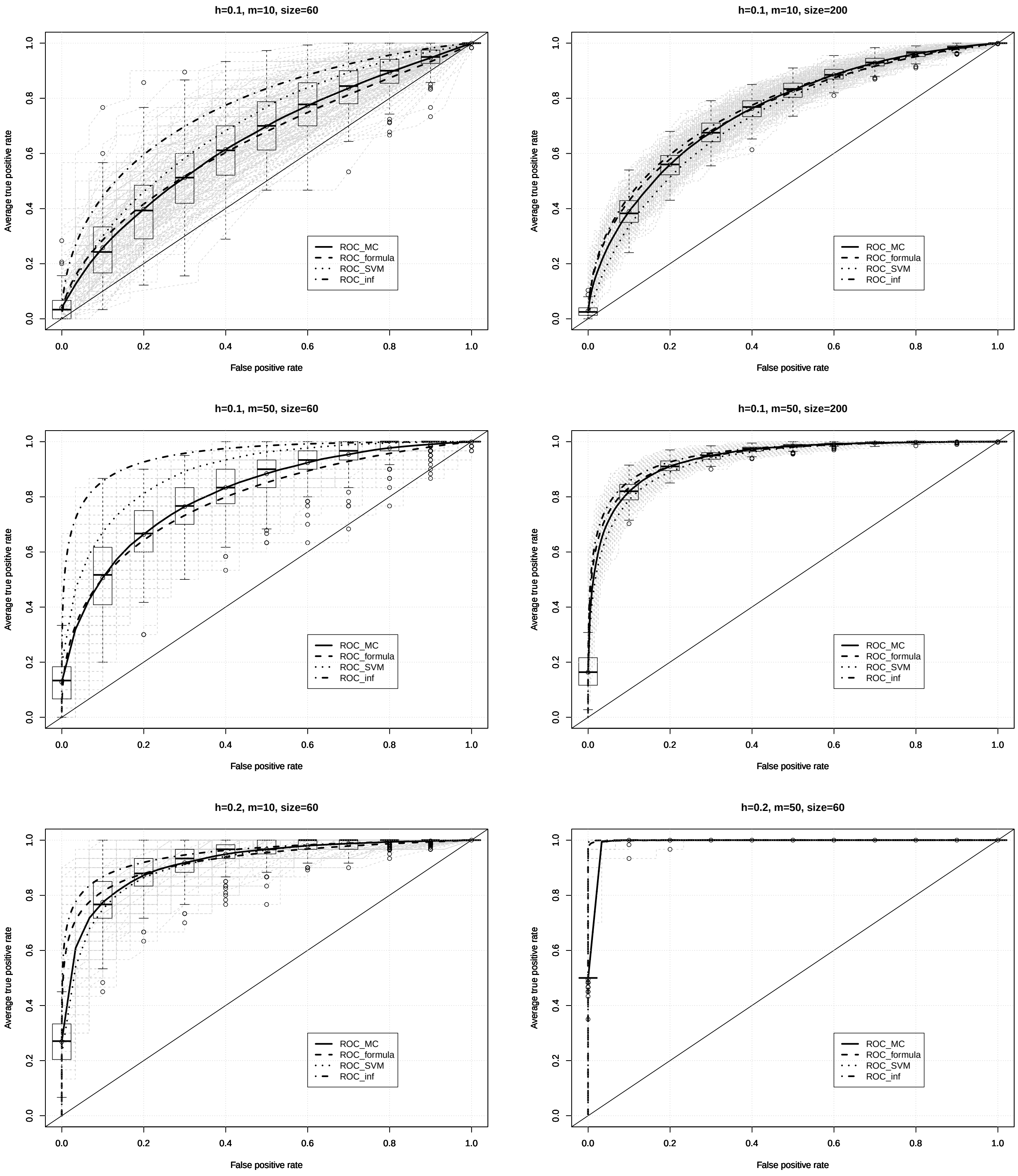

Figure 1: ROC curve for optimal classification, linear classification, Monte Carlo simulation and SVM, the shade is the ROC curve for each simulation. $\alpha = 0.1$, $\rho = 1$

Table 1: Performance of Optimal and Linear classifiers: The values of $A\hat{UC}(n)$ and $A\hat{UC}(n)$MC are close to each other for various model specifications. Here, $\theta_1 = 0.3, h = \theta_1 - \theta_2$, $Size = 2n$ (n for C_1, n for C_2), m is the number of independent SNPs, $\alpha = 0.01$ is the significant level for Wald tests in Section 2.3, and $\rho = 1$ is the percentage of the significant SNPs.

h	m	Size	$A\hat{UC}(\infty)$	$A\hat{UC}(n)$	$A\hat{UC}(n)$MC	$B\hat{i}as$
0.01	10	60	0.5276	0.5021	0.5032	0.0011
0.01	10	200	0.5276	0.5022	0.5022	0
0.01	10	400	0.5276	0.5024	0.5016	-0.0008
0.01	50	60	0.5616	0.5047	0.5183	0.0136
0.01	50	200	0.5616	0.505	0.5111	0.0061
0.01	50	400	0.5616	0.5054	0.5079	0.0025
0.01	200	60	0.6218	0.5094	0.5373	0.0279
0.01	200	200	0.6218	0.5099	0.5217	0.0118
0.01	200	400	0.6218	0.5107	0.5169	0.0062
0.05	10	60	0.6386	0.5171	0.5122	-0.0049
0.05	10	200	0.6386	0.5292	0.5288	-0.0004
0.05	10	400	0.6386	0.5442	0.5439	-0.0003
0.05	50	60	0.7861	0.5382	0.5426	0.0044
0.05	50	200	0.7861	0.565	0.5752	0.0102
0.05	50	400	0.7861	0.5979	0.6197	0.0218
0.05	200	60	0.9436	0.5761	0.5864	0.0103
0.05	200	200	0.9436	0.6283	0.6585	0.0302
0.05	200	400	0.9436	0.6901	0.7367	0.0466
0.2	10	60	0.9488	0.8594	0.8746	0.0152
0.2	10	200	0.9488	0.9471	0.9452	-0.0019
0.2	10	400	0.9488	0.9488	0.9464	-0.0024
0.2	50	60	0.9999	0.992	0.9944	0.0024
0.2	50	200	0.9999	0.9999	0.9999	0
0.2	50	400	0.9999	0.9999	0.9999	0
0.2	200	60	1	1	1	0
0.2	200	200	1	1	1	0
0.2	200	400	1	1	1	0

References

BILLINGSELY, P. (1995) *Probability and Measure*. Wiley, New York.

LIU,X., WANG,Y., REKHAYA,R., and SRIRAM,T.N. (2012) Sample size determination for classifiers based on single-nucleotide polymorphisms. *Biostat.*, **13**, 217-227
